# Building Haplotype‐Resolved 3D Genome Maps of Chicken Skeletal Muscle

**DOI:** 10.1002/advs.202305706

**Published:** 2024-04-06

**Authors:** Jing Li, Yu Lin, Diyan Li, Mengnan He, Hua Kui, Jingyi Bai, Ziyu Chen, Yuwei Gou, Jiaman Zhang, Tao Wang, Qianzi Tang, Fanli Kong, Long Jin, Mingzhou Li

**Affiliations:** ^1^ State Key Laboratory of Swine and Poultry Breeding Industry College of Animal Science and Technology Sichuan Agricultural University Chengdu 611130 China; ^2^ School of Pharmacy Chengdu University Chengdu 610106 China; ^3^ Wildlife Conservation Research Department Chengdu Research Base of Giant Panda Breeding Chengdu 610057 China; ^4^ College of Life Science Sichuan Agricultural University Ya'an 625014 China

**Keywords:** chicken, Hi‐C, homolog, sequence variations, skeletal muscle development

## Abstract

Haplotype‐resolved 3D chromatin architecture related to allelic differences in avian skeletal muscle development has not been addressed so far, although chicken husbandry for meat consumption has been prevalent feature of cultures on every continent for more than thousands of years. Here, high‐resolution Hi‐C diploid maps (1.2‐kb maximum resolution) are generated for skeletal muscle tissues in chicken across three developmental stages (embryonic day 15 to day 30 post‐hatching). The sequence features governing spatial arrangement of chromosomes and characterize homolog pairing in the nucleus, are identified. Multi‐scale characterization of chromatin reorganization between stages from myogenesis in the fetus to myofiber hypertrophy after hatching show concordant changes in transcriptional regulation by relevant signaling pathways. Further interrogation of parent‐of‐origin‐specific chromatin conformation supported that genomic imprinting is absent in birds. This study also reveals promoter‐enhancer interaction (PEI) differences between broiler and layer haplotypes in skeletal muscle development‐related genes are related to genetic variation between breeds, however, only a minority of breed‐specific variations likely contribute to phenotypic divergence in skeletal muscle potentially via allelic PEI rewiring. Beyond defining the haplotype‐specific 3D chromatin architecture in chicken, this study provides a rich resource for investigating allelic regulatory divergence among chicken breeds.

## Graphical abstract

Creating diploid 3D genome maps in animals poses a significant challenge. Here, high‐resolution Hi‐C diploid maps (1.2‐kb maximum resolution) are successfully produced by examining skeletal muscle tissues in hybrid chickens across pre‐ and post‐hatching stages. This research unraveles the dynamic haplotype‐specific chromatin conformation at multiple scales. These findings offer a data‐rich resource that can be leveraged for a more in‐depth exploration of allelical transcriptional regulation.

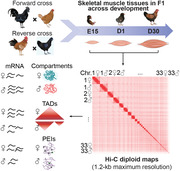

## 1 Introduction

Skeletal muscle tissues (SMTs) represent the largest tissues (by weight) in the animal body and play crucial roles in body movements, posture, and the protection and maintenance of internal organs. Moreover, SMTs hold significant economic value as agricultural animal products.^[^ ^1^ ^]^ The development and growth of SMTs involve intricate and multistep processes governed by tightly coordinated regulatory networks influenced by genome topology. Over the past few decades, substantial efforts have expanded our understanding of the factors governing the prenatal ontogeny of SMTs, as well as the postnatal hypertrophic growth of SMTs and their response to functional maintenance and metabolic homeostasis.^[^ ^1^, ^2^ ^]^ Notably, numerous key regulatory circuits associated with myogenic hyperplasia and hypertrophy have been identified, including myogenic regulatory factors (e.g., MRF4, MyoD, and MyoG),^[^ ^3^ ^]^ transcription factors (e.g., Pax‐3 and ‐7, STAT3),^[^ ^4^ ^]^ growth factors (e.g., IGF, TGF, and GH) and their respective receptors,^[^ ^5^ ^]^ as well as protein kinases (e.g., MAPK, PI3K, and JAK).^[^ ^6^ ^]^

3D chromatin architecture plays a pivotal role in transcriptional regulation,^[^ ^7^ ^]^ undergoing rapid reorganizations to facilitate temporal dynamic interactions within gene regulatory networks. These networks include chromosome territories, compartments, and topologically associating domains (TADs), as well as long‐range interactions between promoters and enhancers (PEIs).^[^ ^8^ ^]^ However, conventional omics data generally fails to distinguish between the two alleles of the diploid genome in somatic cells, and previous studies often infer average chromatin interactions across diploid genomes without considering the unequal contributions of homologous chromosomes (homologs) to cellular function. Understanding the spatial organization of diploid genomes in birds and unraveling the reprogramming of hierarchical chromatin architectures that drive allelically transcriptomic divergence in somatic tissues during development and growth has been long overdue.

The domestic chicken (*Gallus gallus domesticus*) holds immense agricultural importance and serves as a valuable organism for developmental biology due to its manipulability during embryological studies and easy accessibility.^[^ ^9^ ^]^ Over the course of domestication from jungle fowl (*Gallus gallus*) and subsequent selective breeding, significant phenotypic changes have arisen in terms of morphology, physiology, and behavior.^[^ ^10^ ^]^ Domestic chickens have primarily been bred for two purposes: egg‐laying (layer) and meat production (broiler).^[^ ^11^ ^]^ The focused selection by humans on specialized commercial populations, coupled with modern breeding techniques, has yielded remarkable success in enhancing productivity.^[^ ^12^ ^]^ Specialized commercial populations of domestic chickens exhibit substantial sequence diversity, providing ample segregating sites for distinguishing between the two alleles. Moreover, the relatively small genome size of the chicken (≈1 Gb) facilitates the attainment of sufficient sequence coverage, ensuring a reasonable power to characterize the 3D nuclear organization of diploid genomes and shed light on the spatial organization of homologs.

To investigate the haplotype‐resolved chromatin architecture involved in transcriptional regulation throughout the development and growth of SMTs, we employed hybrid chickens generated through reciprocal crosses of highly specialized commercial populations of broiler and layer chickens. The hybrid chicken families enabled us to construct chromosome‐level haplotypes according to their genotypes. Then, we reconstructed the haplotype‐resolved 3D structures of 12 diploid genomes for the hybrid chicken SMTs using ultra‐deep in situ high‐throughput chromatin conformation capture (Hi‐C) sequencing across three representative pre‐ and post‐natal stages. In this way, we could survey the diversity in chromatin architecture across parental haplotypes within the same cellular microenvironment, eliminating statistical errors caused by environmental and individual variability. These Hi‐C maps effectively reveal features of the spatial organization of homologs and enable systematic exploration of the developmentally dynamic alterations, as well as allele‐specific differences based on parent of origin and parental breed, in chromatin conformation across multiple scales. Additionally, we conducted an allele‐specific survey to examine the effects of sequence variations between parental breeds (i.e., broiler vs layer) on the allelic reorganization of PEIs. These reorganizations were found to be moderately associated with allelic imbalances in gene expression within the F1 hybrids.

## 2 Results

### 2.1 Building a Haplotype‐Resolved 3D Genome Map of Chicken Skeletal Muscle

**Figure 1:**
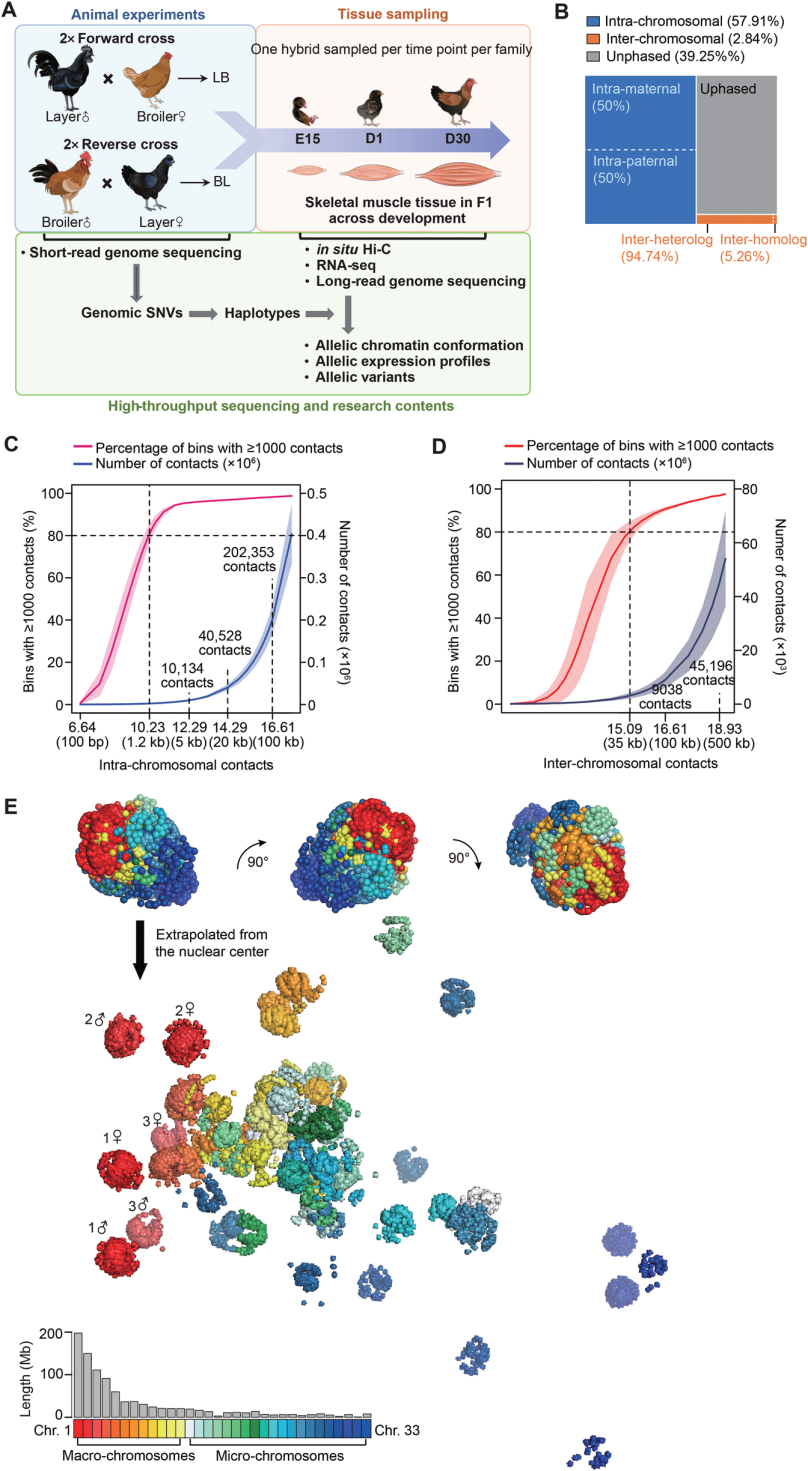
Experimental design and construction of diploid 3D genome maps for chicken skeletal muscle tissue. A) The experimental design of this study aimed to elucidate the regulatory roles of chromatin conformation in gene transcription regulation, as depicted in the schematic representation. B) Allele assignment of in situ Hi‐C contacts in F1 hybrid chickens. A total of approximately 3.54 billion valid Hi‐C contacts were generated for each of the 12 hybrid chicken samples. C,D) Estimation of resolution for haplotype‐resolved intra‐chromosomal (C) and inter‐chromosomal (D) Hi‐C maps. The resolution of the Hi‐C maps refers to the smallest bin size where at least 80% of bins contain a minimum of 1000 reads, ensuring reliable discernment of local features. The data are presented as the mean ± SD of the 24 haplotypes. The mean numbers of contacts at resolutions of 5 kb, 20 kb, and 100 kb are displayed. E) 3D genome structure of a hybrid chicken skeletal muscle sample (BL2‐1) at the E15 stage, constructed using normalized intra‐chromosomal (at 20 kb resolution) and inter‐chromosomal (at 500 kb resolution) contact matrices with miniMDS and the PYMOL program. Upper: an overall view from three different angles. Lower: the structures of autosome pairs. The haplotypes of three pairs of autosomes were marked as examples. Bottom: the lengths of autosomes are indicated with the bar plot.

To explore the dynamic patterns of 3D genome architecture of skeletal muscle tissues (SMTs) during the growth and development of diploid chicken, we carried out reciprocal crosses between Chinese commercial broiler and layer populations, using of two families of Broiler (♂) × Layer (♀), and two families of Layer (♂) × Broiler (♀) (**Figure** **1**A). Whole genome sequencing was then conducted using the two parents and three female F1 hybrid progeny from each of the four families (42.39× coverage for each individual, *n* = 20; Figure S1, Supporting Information). In situ Hi‐C was also conducted for F1 hybrid samples of calf and thigh muscle collected at embryonic day 15 (E15), hatching (D1), and 30 days after hatching (D30) (Figure S2A, Supporting Information), which generated a total of ≈42.51 billion valid Hi‐C contacts (≈3.54 billion per F1 hybrid, *n* = 12) (Figure S2A, Supporting Information).

Using trio‐based genomic and Hi‐C data from F1 hybrids, we constructed the chromosome‐scale haplotypes for each F1 hybrid, which captured 98.00% of all heterozygous single nucleotide variants (SNVs; ≈4.42 per kb) (Figure S3, Supporting Information). SNV phasing was then integrated with local imputation to generate diploid Hi‐C maps for all 12 SMT samples (Figure S2B,C, Supporting Information). In total, ≈24.57 billion valid Hi‐C contacts (≈60% of all valid Hi‐C contacts and ≈1.02 billion per haplotype) were phased (Figure 1B; Figure S2D, Supporting Information), ≈95.12% of which occurred within chromosome and ≈4.62% were between homologs, leaving ≈0.26% connecting non‐homologous chromosomes (i.e., heterologs), ultimately resulting in the high resolution of 1.2 kb for intra‐chromosomal Hi‐C maps and 35 kb for inter‐chromosomal Hi‐C maps (Figure 1C,D).

We also generated haplotype‐resolved transcriptional profiles for the corresponding F1 hybrid samples using the RNA‐seq data (Figure S4A–C, Supporting Information). Allelome.PRO^[^ ^13^ ^]^ was used to estimate the allelic expression of ≈8527 protein coding genes (54.53% of the autosomal genes) that were covered by informative SNVs and had evident transcription above the threshold of 0.5 transcripts per million (TPM) in at least one sample (Figure S4D,E, Supporting Information). These multi‐omic data from this hybrid chicken system enabled systematic investigation of allelic chromatin architecture and related transcriptional regulation.

### 2.2 Chromosomal Distribution and Homolog Pairing in the Chicken 3D Nucleus

We next reconstructed the 3D genome architecture for each of the 12 SMTs based on their haplotype‐resolved Hi‐C maps at 20 kb intra‐chromosomal and 500 kb inter‐chromosomal resolution (Figure 1E). Consistent with previous findings in mammals using bulk or single‐cell Hi‐C data,^[^ ^14^ ^]^ uneven nucleotide distribution was the most prominent characteristic of the 3D genome in somatic tissue of chicken (Figure S5A–G, Supporting Information).

We observed that some sequence‐dependent features of chromosomes were correlated with their 3D spatial arrangement in the nucleus (Figures S6 and S7, Supporting Information). Compared with the 12 macro‐chromosomes (which ranged from 20.20 to 197.61 Mb), the 20 micro‐chromosomes (0.73 to 19.17 Mb) had elevated GC content and higher gene density, but relatively limited repeat sequence regions (Figure S6A, Supporting Information), as shown previously,^[^ ^15^ ^]^ and exhibited relatively higher ratios of inter‐chromosomal contacts (Spearman's *r* = −0.96, *P* < 2.2 × 10^−16^; Figure S5H, Supporting Information). In the nucleus, chromosomes of similar length (e.g., Chromosomes 1 to 5) clustered to form bulk “chromosomal territories” (Figure S5I, Supporting Information). Macrochromosomes and intermediate‐sized microchromosomes occupied internal and external nucleus, while small microchromosomes (shorter than 10 Mb) were predominately located at the nuclear periphery except for Chromosomes 21 and 24 (Figure S6B, Supporting Information). We also noted that homolog pairs were nearly equidistant from the center of the nucleus (Spearman's *r* = 0.83–0.94, *P* < 2.2 × 10^−16^; Figure S5J, Supporting Information), and were generally located in closer spatial proximity than heterologs (Figure S5K,L, Supporting Information).

**Figure 2:**
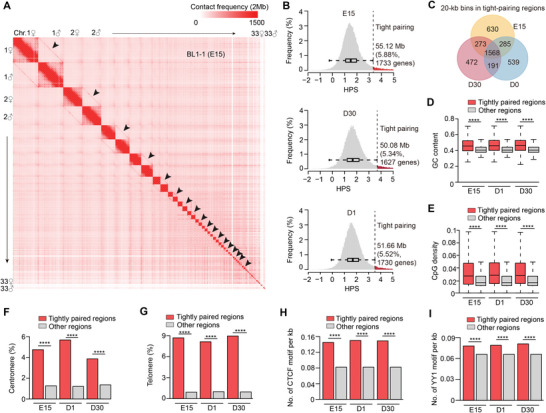
Characteristics of homolog pairing in hybrid chicken samples. A) The close spatial localization of homologous chromosome pairs is highlighted by diagonal lines (black arrows) alongside intra‐chromosomal interaction squares that display relatively intense chromatin interactions between homologs on the Hi‐C map. This figure presents data from a sample (BL1‐1) at the E15 stage. B) The identification of tightly paired regions in the skeletal muscle tissue of hybrid chickens at the three developmental stages is based on the distribution of the homolog pairing score (HPS). Tightly‐paired loci are defined as those with an HPS above Q3 + 1.5 × IQR. C) The Venn plot illustrates the overlap of tightly paired genomic bins (at 20‐kb resolution) identified at the three developmental stages. Approximately 56.89–62.62% of these bins are shared across all three stages. D–I) Analysis of sequence features in the tightly paired regions. These regions exhibit significantly higher GC contents (D), an increased presence of CpG islands (E), more centromere sequences (F), more telomere sequences (G), and a greater abundance of CTCF (H) and YY1 (I) binding sites compared to other genomic regions. The *P* values were calculated using the Wilcoxon rank‐sum test in (D,E) and Fisher's exact test in (F–I). ^**^0.001< *p* <0.01; ^***^ *p* <0.001.

In some organisms, such as *Drosophila*, the entire genome pairs in somatic tissues (known as ‘homolog pairing’), whereas little homolog pairing outside the germline has been documented in most others including birds.^[^ ^16^ ^]^ With the aid of ≈99.55 million inter‐chromosomal contacts detected in the 12 hybrid F1 samples, we observed that homologs were aligned throughout the chicken genome, with clear signatures of inter‐homolog interactions observable in the Hi‐C maps (**Figure** **2**A; Figure S8A,B, Supporting Information). We further quantified the intensity of homolog co‐localization using homolog pairing score (HPS)^[^ ^17^ ^]^ and identified 50.08–55.12 Mb tightly‐pairing regions (5.21–5.74% of the genome) at each developmental stage (Figure 2B), 31.36 Mb of which were shared among stages (Figure 2C). Similar to the findings in fruit flies and mammals,^[^ ^18^ ^]^ these tightly paired regions within the chicken genome were likely to occur in centromeres and telomeres which are rich in repeated sequences,^[^ ^19^ ^]^ and had relatively higher GC contents, more CpG islands, and greater density of binding sites for chromatin structural proteins (i.e., CTCF and YY1) than other regions (Figure 2D–I; Figure S8C,D, Supporting Information). These results thus revealed that homolog pairing was strongly correlated with genetic/epigenetic features of the genome.

### 2.3 Haplotype‐Resolved Hierarchical 3D Chromatin Structures of Chicken Genome

To facilitate comparison of 3D genome features between haplotypes, we subsequently identified hierarchical chromatin architectures. Based on the haplotype‐resolved Hi‐C maps at 20‐kb resolution, we first defined the accessible, actively transcribed A compartments (50.11–53.31% of the genome; 481.62–512.37 Mb), while the remaining regions were designated as B compartments (Figure S9, Supporting Information). At a finer scale, the haplotype genomes could be partitioned into 1656–1720 TADs (Figure S10A, Supporting Information), with a median size of ≈420 kb and accounting for ≈83.29% of the genome (Figure S10B, Supporting Information). TAD boundaries were enriched with CpG islands, transcriptional start sites (TSS), and long terminal repeats (LTRs), but depleted for other transposable elements (TEs), including short interspersed nuclear elements (SINEs) and DNA transposons (Figure S10C–G, Supporting Information), as reported in other organisms in previous studies.^[^ ^20^ ^]^

Using the PSYCHIC algorithm^[^ ^21^ ^]^ with 5‐kb resolution Hi‐C maps, we further identified 17,527–20,634 PEIs in each of the 24 haplotypes, with a median bridging distance of 80 kb (ranging from 25 kb to 1 Mb) (Figure S11A,B, Supporting Information). The vast majority (≈85.44%) of PEIs were limited to regions within TADs (Figure S11C, Supporting Information). We also found that promoters mainly interacted with distal enhancers (≈19.99%) or bypassed at least one enhancer (≈55.35%), while the remaining promoters interacted with the nearest enhancers (Figure S11D, Supporting Information). Supporting the additive effects of multiple enhancers on target gene transcription,^[^ ^22^ ^]^ autosomal genes interacting with one or more enhancers (≈37.34%) were expressed at higher levels than those showing no or fewer enhancer interactions (Figure S11E,F, Supporting Information). Merging the PEIs identified at the same developmental stage allowed us to assemble a set of PEIs common to all samples from the same stage (*n* = 40,844 (E15), 39,410 (D1), and 37,082 (D30)). To quantitatively evaluate the regulatory effects of enhancers on individual genes, we then calculated the regulatory potential score (RPS, a spatial proximity‐based index)^[^ ^23^ ^]^ for each gene to account for the combined regulatory effects of multiple enhancers on a given gene. As expected, larger RPS was associated with higher expression levels (Figure S11G, Supporting Information), thus supporting that enhancers actively regulate gene expression.^[^ ^24^ ^]^

It was noted that closer examination of Hi‐C maps and hierarchical layers of chromatin conformation (i.e., compartments, TADs, PEIs) collectively revealed that haplotypes had greater differences in 3D genome architecture between stages than between parents of different origin or between different breeds at the same stage (**Table** **1**; Figure S12, Supporting Information). These results were recapitulated by allelic expression data, implying that changes in chromatin architecture might facilitate concomitant shifts in transcriptional activity.

**Table 1:** Summary of allelic chromatin reorganization at multiple scales.

Haplotype‐resolved characteristics	Measurements	Neighboring stages		Parental breeds			Parents of origin		
		E15 vs. D1	D1 vs. D30	E15	D1	D30	E15	D1	D30
Hi‐C contact map	Similarity (GenomeDISCO)	0.955	0.961	0.973	0.972	0.969	0.973	0.972	0.969
Compartment	A/B switched regions^a)^ [Mb]	30.86	19.28	1.00	0.92	1.08	1.64	0.98	0.64
	A/B variable regions^b)^ [Mb]	71.46	40.16	10.18	6.46	5.90	5.08	4.18	3.96
TAD	No. of shifted boundaries	37	15	–	–	–	–	–	–
PEI	No. of genes with differential RPS^c)^	2116	1973	334	302	280	308	301	308

^a)^ A/B switched regions were defined as segments exhibiting distinct compartment status between haplotypes (from A [positive value of A‐B index] to B [negative value of A‐B index], or from B to A);

^b)^ A/B variable regions were defined as segments showing the same compartment status but differential A‐B index (|ΔA‐B index| >1 [between neighboring stages] or |ΔA‐B index| >0.3 [between breeds or parents of origin] and *p* <0.05, paired Student's *t*‐test);

^c)^ Significantly differential RPS were determined by |ΔRPS| (between neighboring stages: |ΔRPS| >3; between breeds or parents of origin: |ΔRPS| >0.3) and paired Student's *t*‐test (*p* <0.05).

### 2.4 Allelically Dynamic Chromatin Architecture During Development and Growth of the Chicken Skeletal Muscle

Chicken and other amniotes share a multi‐step process of muscle growth and development that primarily consists of the prenatal hyperplasia followed by postnatal hypertrophy of heterogeneous myofibers. We first explored how haplotype‐resolved chromatin architecture appears to shift across the three representative pre‐ and postnatal stages of skeletal muscle development.

#### 2.4.1 Compartmental Rearrangements

**Figure 3:**
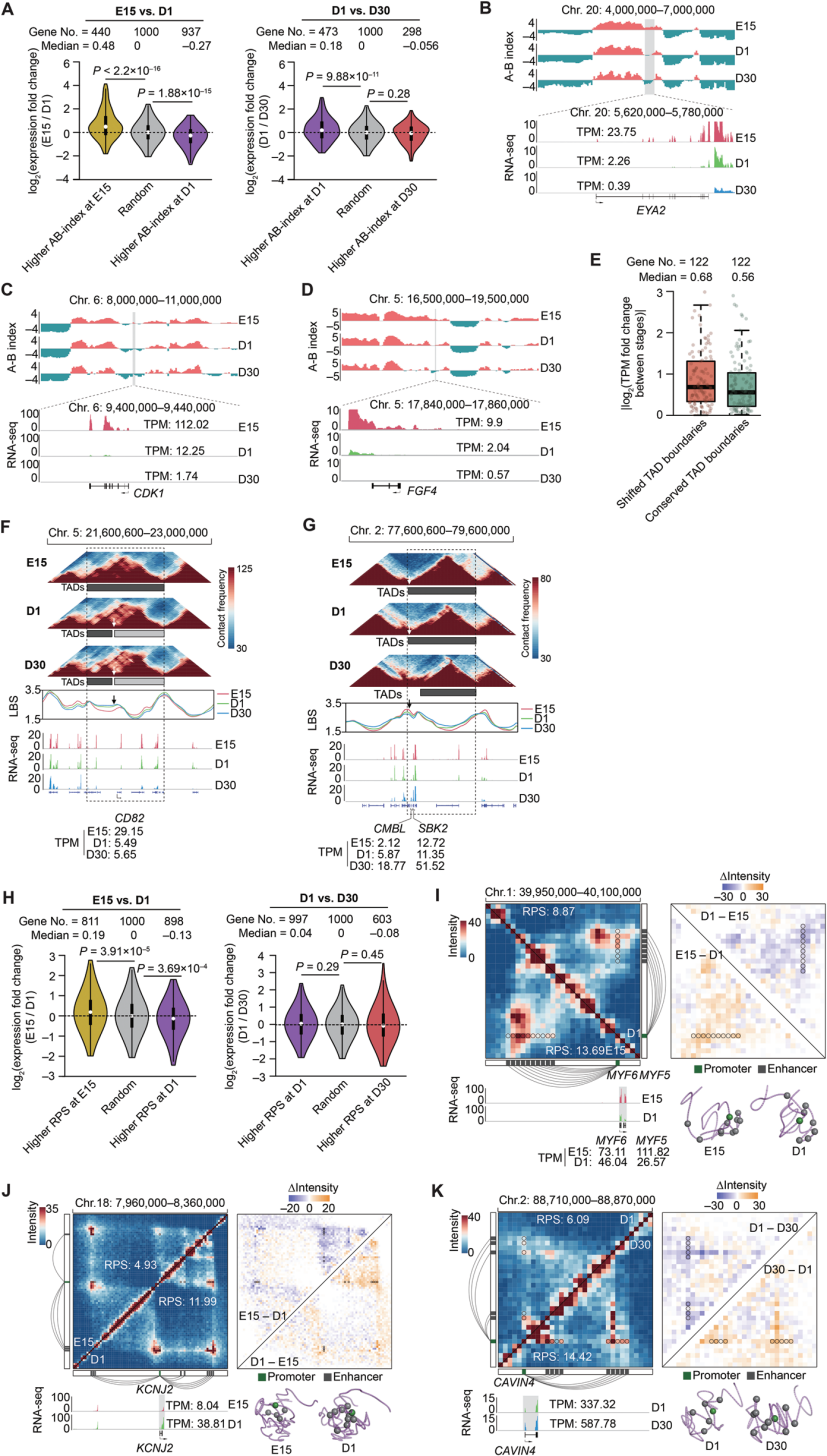
Characteristics of differential chromatin architectures identified between neighboring developmental stages in chicken skeletal muscle tissue. A) Expression differences are observed between neighboring stages for genes located in stage‐restricted, more active compartments, including A/B switched and A/B variable compartments. B–D) Examples of genes with switched compartments and differential expression across skeletal muscle development are *EYA2* (B), *CDK1* (C), and *FGF4* (D), all of which play important roles in myogenesis and skeletal muscle growth. E) Comparison of differential expression levels for genes near shifted and conserved TAD boundaries between developmental stages. A total of 122 genes were located at shifted boundaries and had allelic expression data. An equal number of genes were randomly selected from conserved boundaries for 100,000 iterations. F,G) Stage‐specific TAD boundaries adjacent to genes responsible for cell differentiation and muscle development. An E15‐specific TAD boundary loss overlaps with *CD82* (F), while a D30‐specific TAD boundary shift affects *CMBL* and *SBK2* (G). These genes exhibit corresponding changes in expression across development. The figure includes Hi‐C maps (at 20‐kb resolution), TAD boundaries, local boundary scores (LBS), RNA‐seq signals in the corresponding region, as well as structures and transcripts per million (TPM) values of the putatively affected genes. Black dashed boxes indicate TAD boundary changes during development. H) Expression differences are observed for genes with significantly differential RPS between neighboring stages. I–K) Rewiring of PEIs is observed for three representative genes with differential RPS between neighboring stages: *MYF5* and *MYF6* (genes enriched in the “embryonic skeletal muscle system morphogenesis” gene ontology term and sharing the promoter bin; I), *KCNJ2* (gene enriched in the “cGMP‐PKG signaling pathway” and “actin filament‐based process”; J), and *CAVIN4* (gene enriched in “muscle structure development”; K). The figure presents Hi‐C maps (upper left), heatmaps of interaction intensity differences (upper right), gene expression levels (lower left), and 3D structural models (lower right) of the corresponding genomic regions. Promoters (green squares), enhancers (grey squares), and PEIs (connecting lines) are displayed alongside the Hi‐C maps. The significance of the differences in (A) and (H) was evaluated using the Wilcoxon rank‐sum test.

In accordance with the more prominent physiological and morphological changes in SMTs that occur between prenatal myogenesis and postnatal hypertrophy of myofibers, we also observed that compartmental rearrangements (including A/B switches and A/B variables) were more dramatic from E15 to D1 (102.32 Mb, 10.65% of the genome) than those occurring between D1 and D30 (59.44 Mb, 6.19% of the genome) (Table 1; Data S1, Supporting Information). This finding was further supported by the relatively higher allelic variation in transcription between E15 and D1 (Pearson's *r* = 0.889, *P* < 2.2 × 10^−16^) than between D1 and D30 (Pearson's *r* = 0.915, *P* < 2.2 × 10^−16^). Genes located in more active compartments showed generally higher expression in corresponding stage (**Figure** **3**A) and were mainly involved in biological processes related to myogenesis and postnatal hypertrophy of myofibers (Figure S13, Supporting Information).^[^ ^25^ ^]^

Typically, genes associated with macromolecule catabolism and fat cell differentiation had a more active compartmental status at D1 compared to E15 (Figure S13B, Supporting Information). This finding implied that SMTs displayed more dynamic metabolic, energy‐ and nutrient‐consuming processes after hatching. Alternatively, genes related to ‘regulation of mesenchymal cell proliferation’ generally had a more repressive compartmental status in D30 compared with D1 (Figure S13C, Supporting Information), which was consistent with observations that suggest myogenic hyperplasia gradually decreases during postnatal SMT maturation. Among 33 genes that are abundantly expressed in myocytes during embryonic/fetal myogenesis in vertebrates,^[^ ^4^ ^]^ 10 (30.30%) genes shifted compartmental status from active at E15 to repressive at D30 (Figure S14A, Supporting Information), such as *EYA2* (Figure 3B), a premyogenic mesoderm factor gene essential for hypaxial muscle development during embryonic stages in mice.^[^ ^26^ ^]^

Additionally, we found that 22 (64.71%) of 34 genes from an a priori gene set^[^ ^1a^ ^]^ exhibited stage‐dependent patterns of compartmentalization (Figure S14B, Supporting Information). By contrast, very few of these genes encoding protein kinases, growth factors, and growth factor receptors crucial for SMT development and biomass production in farm animals (e.g.*, AKT1*, *CDK9*, *GH*, *IGF1R*, *TGFB1*, and *TGFB2*)^[^ ^1a^ ^]^ maintained an active compartment status across all three developmental stages. For example, the cell cycle regulator *CDK1*,^[^ ^27^ ^]^ and an embryonic limb musculature development factor *FGF4*,^[^ ^28^ ^]^ both had more active compartment status and higher transcription levels prior to hatching (Figure 3C,D). These results thus revealed that genes required for SMT development and growth had characteristic covariance between compartment status and transcriptional regulation.

#### 2.4.2 Variable TAD Boundaries

Although TAD boundaries were largely invariant across stages (Pearson's *r* of LBS = 0.941, *P* <2.2 × 10^−16^), we identified 37 TADs that shifted boundaries between stages E15 and D1 and 15 TADs with shifted boundaries between D1 and D30 (Table 1; Data S2, Supporting Information). Genes near stage‐specific boundaries (94 genes for E15 to D1 and 28 genes for D1 to D30) showed relatively greater changes in their expression levels than those near boundaries conserved between stages (Figure 3E). These results highlighted the roles of TADs in establishing a local regulatory context for genes.

For example, *CD82*, a signature of muscle stem cells essential for myogenic progenitor activation and differentiation,^[^ ^29^ ^]^ was located within a TAD at E15 but shifted to the boundary at D1 and D30 (Figure 3F). Consistent with the boundary loss at E15, *CD82* had more local interactions and higher expression at E15 than at D1 or D30 (Figure 3F), a pattern likely related to its more active role in myogenesis before hatching. In contrast, *CMBL* (a key regulator of myoblast differentiation)^[^ ^30^ ^]^ and *SBK2* (essential for sarcomere integrity)^[^ ^31^ ^]^ were both embedded in a TAD boundary at E15 and D1, and had more local interactions and higher expression levels at D30, at which point these genes were detected outside of the TADs (Figure 3G), which aligns well with their roles in rapid growth and functional maintenance of SMTs in the post‐hatching D30 stage. These results highlight the possible role of TADs in shifting patterns of transcriptional regulation through modulation of local chromatin interactions during SMT development and growth.

#### 2.4.3 PEI Rewiring

In addition to changes in TAD boundaries and compartment status, we observed extensive rewiring of PEIs between adjacent developmental stages. For instance, 2116 genes had significantly different RPS (|∆RPS| > 3 and *P* < 0.05, paired Student’s *t*‐test; Table 1) between E15 and D1, while 1973 had different RPS between D1 and D30 (Data S3, Supporting Information), the majority of which also displayed differential transcription between stages (Figure 3H).

From E15 to D1, genes with higher RPS at E15 (*n* = 1042) were mainly involved in processes related to embryonic muscle morphogenesis, such as ‘skeletal muscle cell differentiation’, ‘mesoderm formation’, and ‘cellular response to fibroblast growth factor stimulus’ (Figure S15A, Supporting Information). In contrast, genes with higher RPS at D1 (*n* = 1074) were primarily involved in processes of muscle growth and contraction, including ‘transmembrane receptor protein tyrosine kinase signaling pathway’ (which is closely linked to cell proliferation during muscle growth), ‘regulation of myosin‐light‐chain‐phosphatase activity’, ‘focal adhesion’, ‘membrane repolarization during action potential’, ‘cGPM‐PKG signaling pathway’ (which mediates the regulation of myocyte relaxation and contraction), among others (Figure S15B, Supporting Information).

From D1 to D30, genes with higher RPS at D1 (*n* = 1230) were mainly enriched in pathways related to transmembrane receptor protein tyrosine kinase signaling (including ‘Ras protein signal transduction’, ‘Hippo signaling pathway’, and ‘BMP signaling pathway’) and muscle development associated processes (e.g., ‘regulation of fibroblast growth factor receptor signaling pathway’, ‘positive regulation of collagen metabolic process’, and ‘fat cell differentiation’) (Figure S15C, Supporting Information), which generally supports the notion that myofiber numbers are almost fully determined (i.e., secondary myogenesis is almost complete) by hatching,^[^ ^32^ ^]^ with SMT prepared for rapid hypertrophy of myofibers post‐hatching, and that adipocytes terminally differentiate around hatching to determine intramuscular adipogenesis.^[^ ^33^ ^]^ In contrast with D1, genes with higher RPS at D30 (*n* = 743) were mainly associated with muscle system processes (e.g., ‘muscle contraction’), sarcomere organization, muscle adaptation (e.g., ‘regulation of release of sequestered calcium ion into cytosol by sarcoplasmic reticulum’ and ‘skeletal muscle adaptation’), and immune processes (Figure S15D, Supporting Information). These GO analyses emphasize that the main biological processes active in SMT at D30, a peak stage of skeletal muscle growth, feature hypertrophy, and functional maturation of myofibers with well‐established contractile activity. Representative gene examples with differential RPS between stages are shown in Figure 3I–K and Figure S16, Supporting Information.

Taken together, these results provide a multi‐level characterization of the dynamic chromatin architecture associated with the gradual transition of physiological functions from prenatal development to postnatal growth and maturation in SMT cells.

### 2.5 Parent‐of‐Origin Specific 3D Chromatin Structure Confirms the Absence of Genomic Imprinting in Chicken

**Figure 4:**
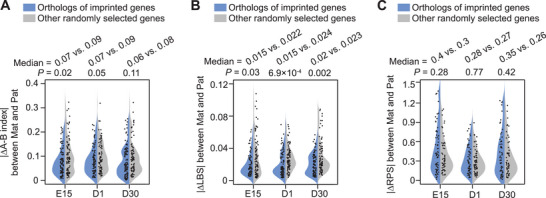
Comparison of absolute values of A‐B index differences (A), local boundary score (LBS) differences (B), and regulatory potential score (RPS) differences (C) between the 106 chicken orthologs of empirical imprinted genes and randomly selected genes (*n* = 106) at E15, D1, and D30 stages. Mat: maternal haplotypes; Pat: paternal haplotypes. The *P* values were calculated using a Wilcoxon rank‐sum test.

Although many studies have shown that genomic imprinting (a phenomenon characterized by parent‐of‐origin‐specific expression)^[^ ^34^ ^]^ is absent in oviparous birds,^[^ ^35^ ^]^ few studies have yet checked for evidence imprinting in the 3D genome architecture of *Gallus gallus domesticus*. In line with previous reports using only allelic expression data,^[^ ^35^, ^36^ ^]^ we found no genes with parent‐of‐origin‐specific patterns of transcription in any of the three stages examined here (Figure S17A, Supporting Information). We next checked for allelic differences in the chromatin architecture of 106 genes in the chicken genome that are orthologous to the 126 known imprinted genes (downloaded from https://geneimprint.com/site/home). Notably, no significantly larger differences in parent‐of‐origin biased compartmentalization, TAD formation, or PEI wiring were identified in any of the genes than others (**Figure** **4**; Figure S17B–D, Data S4, Supporting Information). These results thus confirmed the absence of supporting genomic imprinting in chicken SMT, at least in the three pre‐ and post‐hatching stages tested here.

### 2.6 Allelic Differences in 3D Genome Architecture Between Broiler and Layer Lines

We next investigated potential allelic differences in 3D genome architecture that could lead to imbalanced expression of haplotypes between the two parental breeds, potentially contributing to the dramatic phenotypic divergence of SMT between the layer and broiler populations. In total, 1.16% (11.18 Mb), 0.77% (7.38 Mb), and 0.73% (6.98 Mb) of the genome had variable or switched compartment status between breeds at E15, D1, or D30 stages, respectively (Table 1; Data S5, Supporting Information). Changes in these regions were highly stage‐specific (Figure S18A, Supporting Information) and the allelic bias of the embedded genes showed general agreement between compartmentalization and expression level (Figure S18B, Supporting Information). Remarkably, genes located in more active compartments of broiler haplotypes (*n* = 129, 97, and 96 for E15, D1, and D30, respectively) were mainly involved in processes related to ‘cardiac muscle cell development’, ‘endochondral ossification’, and ‘muscle system process’ (Figure S18C, Supporting Information), implying strong selection for skeletal muscle mass in broilers, and thus resulting in a faster growth rate and larger body size than that of the layer phenotype.

**Figure 5:**
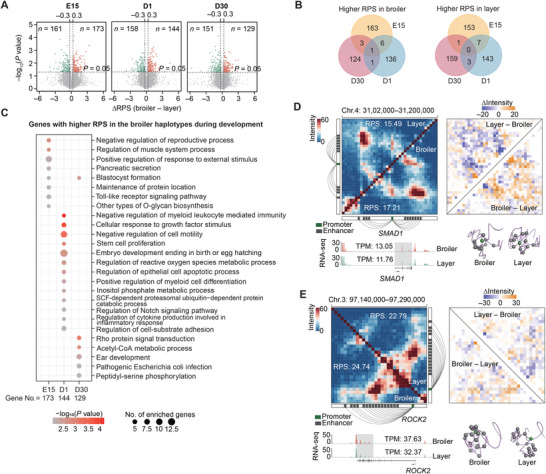
Characteristics of differential RPS genes between parental breeds in the hybrid chickens. A) Volcano plots illustrating genes with statistically different RPS (|ΔRPS| > 0.3 and *P* < 0.05, paired Student's *t*‐test) between parental breeds at E15 (left), D1 (middle), and D30 (right) developmental stages. B) Venn plots displaying genes with higher RPS in either broiler (left) or layer haplotypes (right) at the three developmental stages. Gene numbers are labeled on the plots. C) Results of functional enrichment analysis conducted for genes with elevated RPS in broiler haplotypes across the three developmental stages. The top twenty enriched terms are shown. D,E) PEI rewiring of two representative genes with differential RPS between breeds: *SMAD1* (related to ‘response to growth factor’; D) and *ROCK2* (related to ‘Rho protein signal transduction’; E). The figures display the Hi‐C maps (upper left), heatmaps of interaction intensity differences (upper right), gene expression levels (lower left), and 3D structural models (lower right) of the corresponding genomic regions. Promoter regions are denoted by green squares, enhancers by grey squares, and PEIs by connecting lines beside the Hi‐C maps.

No variable TAD boundaries were detected between the two parental breeds at any of the three developmental stages. In total, 334, 302, and 280 genes with differential RPS (|ΔRPS| >0.3 and *P* <0.05, paired Student’s *t*‐test; Table 1) were identified between the broiler and layer haplotypes at E15, D1, and D30 stage, respectively (**Figure** **5**A,B; Data S6, Supporting Information). Among them, several genes exhibited a greater number of potential intrachromosomal interactions in the broiler alleles, suggesting more intense spatial regulatory circuitry, and implying these might be likely candidate alleles associated with phenotypic divergence of SMT between parental breeds, for instance, enhanced hyperplasia resulting in a larger number of myofibers in broilers before hatching, and a greater degree of hypertrophy in myofibers which could lead to greater muscle mass (body weight in marketing age: 2.350 ± 0.089 of broiler versus 1.674 ± 0.053 of layer^[^ ^37^ ^]^ and larger myofibers in broilers after hatching (myofiber diameter in thigh muscle at 28‐day‐old: 25.97 ± 2.66 µm of broiler versus 17.34 ± 1.36 µm of layer).^[^ ^38^ ^]^

More specifically, 173 genes that were mainly associated with ‘regulation of muscle system process’ and ‘toll‐like receptor signaling pathway’ were identified at E15 with higher RPS in the broiler alleles (Figure 5C), which was in agreement with known functions of toll‐like receptors in muscle development.^[^ ^39^ ^]^

In the D1 stage, 144 genes were detected with higher RPS in the broiler alleles which were mainly involved in ‘embryo development ending in birth or egg hatching’, ‘stem cell proliferation’, ‘cellular response to growth factor stimulus’, and ‘regulation of Notch signaling pathway’, the latter of which is a negative regulator of myogenesis,^[^ ^40^ ^]^ among other processes (Figure 5C). Typically, broiler alleles of *SMAD1* had higher RPS and expression levels than corresponding layer alleles, which aligned well with its role as a positive regulator of skeletal muscle mass and myofiber size (Figure 5D).^[^ ^41^ ^]^

In D30 chicks, 129 genes were found with higher RPS in the broiler alleles that were primarily involved in processes contributing to enhanced SMT mass (i.e., SMT with high energy‐consuming and metabolically active hypertrophic myofibers). These processes included ‘acetyl‐CoA metabolism’, which is essential for respiration and energy production,^[^ ^42^ ^]^ and ‘Rho protein signal transduction’, which regulates glucose uptake in SMTs (Figure 5C).^[^ ^43^ ^]^ For example, broiler alleles of *ROCK2* had higher RPS and transcript levels than layer alleles (Figure 5E), which aligned well with its role in myogenic hypertrophy and SMT maturation.^[^ ^44^ ^]^ Other examples of SMT development‐related genes with differential regulation between broiler and layer alleles are shown in Figure S19, Supporting Information.

### 2.7 Effects of Genetic Variation on Allelic PEI Rewiring Between Parental Lines

Since 3D genome organization can be disrupted by genomic rearrangements, we next explored whether and how heritable sequence variations between broiler and layer populations could impact allelic PEI rewiring associated with phenotypic divergence in SMT between parental breeds.

Considering the functional impacts of large insertion and deletions (indels) on 3D genome organization, we generated long‐read sequencing data for the 12 F1 hybrids (≈80.76× coverage per individual, Figure S20A, Supporting Information) using the Oxford Nanopore Technology (ONT) platform to identify allele‐resolved large indels (Figure S20B, Supporting Information). Then, combining these large indels with allelic SNVs and short indels identified in short‐read genome sequencing data, we observed that, as expected, higher sequence divergence (evaluated by identity score; IDS) in promoters or enhancers was linked to greater differences in PEI intensity between broiler and layer alleles (Figure S20C–G, Supporting Information). The results confirmed that allelic variations in promoters and enhancers could indeed drive allelic differences in PEI wiring between broiler and layer lines.

**Figure 6:**
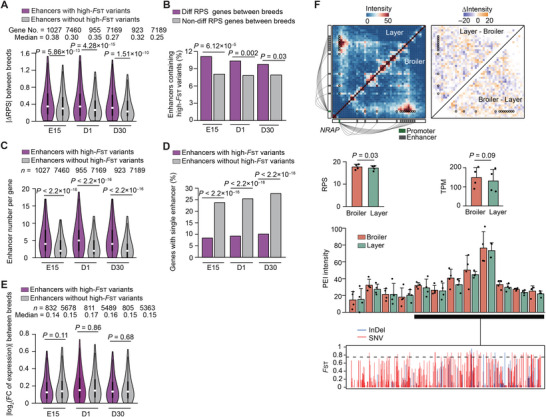
Effects of highly divergent sequences (indicated by high‐*F*_ST_ variants) in enhancer regions between breeds on PEI wiring in the hybrid chickens. A) Genes with enhancers containing high‐*F*_ST_ variants exhibit greater changes in RPS. (A) Comparison of RPS differences for genes with enhancers carrying high‐*F*_ST_ variants versus those lacking them. B) Comparison of enrichment in differential RPS genes for genes with enhancers carrying high‐*F*_ST_ variants versus those lacking them. C)Genes with enhancers containing high‐*F*_ST_ variants have a significantly greater number of enhancers compared to those lacking them. D) Genes with enhancers containing high‐*F*_ST_ variants have a lower number and percentage of genes interacting with a single enhancer compared to those lacking high‐*F*_ST_ variants. E) No significant expression differences between breeds were observed for genes with enhancers containing high‐*F*_ST_ variants compared to those lacking them at the three developmental stages. F) *NRAP*, a differential RPS gene, with enhancers enriched for high‐*F*_ST_ variants, displaying divergent expression between parental breeds. The figure showcases the comparison of Hi‐C maps (upper left), interaction intensity differences (upper right), RPS (middle left), gene expression levels (middle right), and PEI intensities and sequence divergence in the enhancer regions (measured by *F*_ST_ of variants calculated using the genotypes of 24 purebred chickens; lower) between broilers and layers. Promoters (green square), enhancers (grey squares), and PEIs (connecting lines) are displayed beside the Hi‐C maps. The *P* values were calculated using the Wilcoxon rank‐sum test in (A,C,E) and Fisher's exact test in (B,D,F).

Further, we screened high‐*F*_ST_ variants (*F*_ST_ ≥ 0.75) in the F1 hybrids using the genotype data of the 12 broilers and 12 layers, which represented the most divergent sites between breeds, and found that genes carrying high‐*F*_ST_ variants in their enhancer regions displayed significantly higher differences in RPS than genes without high‐*F*_ST_ enhancer variants (**Figure** **6**A). Correspondingly, high‐*F*_ST_ variants were enriched in the enhancers of differential RPS genes between breeds (Figure 6B). However, this trend was not observed in promoter regions (Figure S20H,I, Supporting Information), suggesting that breed‐specific enhancer variants likely play a prominent role in allelic PEI rewiring between the broiler and layer haplotypes.

We then examined if high‐*F*_ST_ enhancer variants were associated with phenotypic divergence in chicken SMT between parental breeds via allelic PEI rewiring. The highest enrichment of genes (*n* = 1379) with high‐*F*_ST_ enhancer variants was found in processes related to SMT development and growth, such as ‘negative regulation of muscle hypertrophy’ and ‘negative regulation of myoblast differentiation’ (Figure S20J, Supporting Information), which supported a link between breed‐specific regulatory variants in SMT development related genes and phenotypic differences between broilers and layers. However, the genes with high‐*F*_ST_ enhancer variants had contacts with significantly more enhancers and showed unexpectedly lower expression differences between breeds than those without high‐*F*_ST_ enhancer variants (Figure 6C–E). This implies that most enhancers with high‐*F*_ST_ variants regulate transcription in a more redundant manner, and thus, among thousands of breed‐specific variants, only a few are likely responsible for SMT phenotypic differences between chicken breeds through disrupting PEI. In one notable example found here, a hotspot of high‐*F*_ST_ variants overlapped with multiple enhancers of *NRAP*, and this gene showed higher RPS as well as a trend of higher expression among broiler alleles compared to layer alleles at D30 (Figure 6F), which could affect its function in myofibril assembly, specifically membrane anchoring of myofibril actin filaments.^[^ ^45^ ^]^

## 3 Discussion

Constructing haplotype‐resolved 3D genome maps in animals remains challenging and a landscape perspective of the dynamics of chromatin architecture during avian development lags far behind that of mammals and other models, although a clear understanding of changes in chromatin organization could provide a valuable resource for studies examining the functional genomics and allelic transcriptional regulation underlying phenotypic variability in birds. Here, with the aid of reciprocal crosses between genetically distinct broiler and layer chicken breeds (≈4.42 heterozygous SNVs per kb in F1 hybrids), we generated diploid Hi‐C maps at a maximum resolution of 1.2 kb for chicken SMT at three representative pre‐ and post‐hatching stages. By combining haplotype‐resolved transcriptomic and genomic variation data, we systematically examined allelic differences in the hierarchical chromatin architecture between developmental stages, parents of origin, and parental breeds, as well as their associations with allelic divergence in transcriptional regulation and breed‐specific sequence variants.

Using the haplotype‐resolved 3D genome maps, we found that the sequence‐dependent features (e.g., gene density, expression status) and chromosome size were primary factors affecting the spatial arrangement of chromatins. This higher‐order organization of the diploid avian genome in somatic tissue is similar to that reported in mammals, such as humans and mice,^[^ ^18^, ^46^ ^]^ supporting the likelihood that these features control chromosomal organization across vertebrate species. Whereas the distinct features of macro‐chromosomes and micro‐chromosomes on the organization of chromosome territories in chicken genome are required further investigation.

Homolog pairing (that is, the genome‐wide co‐localization of homologs in the nucleus) was first described in *Drosophila* nearly a century ago, but is still poorly surveyed in mammals and birds.^[^ ^18^, ^47^ ^]^ Using a similar analytical pipeline to that used in fruit fly^[^ ^17^, ^18^ ^]^ and the high‐resolution diploid Hi‐C maps, we provide the first snapshoot of ‘homolog pairing’ in somatic tissue of chicken, and generate resource for further investigation of the mechanisms responsible for pairing, as well as the functional implications of pairing in gene regulation in individuals originated from genetically divergent parents. Although the signals of homolog pairing were observed in chicken somatic tissues, even only using the Hi‐C interactions phased by the heterozygous SNVs of highest confidence (Figure S21, Supporting Information), further experimental investigation is required to exclude technical and analytic biases that could be potentially confused with bona fide signals from co‐localization of homologs.

This study also characterizes the dynamic changes in hierarchical chromatin architectures between developmental stages at the haplotype level, including compartmental rearrangement, shifting TADs, and PEI rewiring, which are accompanied by concordant allelic transcriptional changes and gradual transition among biological processes during SMT development (i.e., from myogenesis in the fetus to myofiber hypertrophy after hatching).^[^ ^48^ ^]^ Our analysis of PEIs can enrich the annotation of regulatory DNA elements in the chicken genome to facilitate in‐depth functional characterization in future studies. It should be noted that SMT undergoes changes in cell composition during development.^[^ ^33^, ^49^ ^]^ Thus, further investigation is necessary at single‐cell resolution to dissect both 3D chromatin architecture and transcriptional regulation.

Genomic imprinting has been found to be closely associated with parent‐of‐origin‐specific chromatin organization.^[^ ^50^ ^]^ Through sampling the F1 progeny of reciprocal crosses and identifying parent‐of‐origin‐biased 3D chromatin structure, we confirm that genomic imprinting is absent from the genome of SMT cells in chicken, at least in the three pre‐ and post‐hatching stages tested here.

This study reveals that allele‐specific PEI formation in F1 hybrids is correlated with parental breed‐specific sequence variations (i.e., broiler versus layer). Nonetheless, we found that only a few specific genetic variations (such as in enhancer regions) associated with allelic PEI rewiring likely contribute to differences in SMT physiology and phenotype between the specialized broiler (meat‐producing) and layer (egg‐producing) chickens. In contrast to the large chromosome rearrangements leading to cancers^[^ ^51^ ^]^ or severe developmental disorders^[^ ^52^ ^]^ through dramatic 3D genome reorganizations (e.g., TAD shifts), most breed‐specific variations, although probably causing allelic PEI rewiring, had a subtle phenotypic effect. We speculate that this is most likely due to remarkable redundancy in contacts among rewired enhancers carrying high‐*F*_ST_ variants (Figure 6C–E). That is, contacts of multiple enhancers to the same promoter could potentially ensure that local regulatory circuitry is maintained to buffer genetic disruption caused by variants in a single enhancer. Only breed‐specific variant hotspots under ultra‐strong selection and overlapping multiple enhancers of a gene may result in phenotypic changes via transcriptional regulation mediated by PEI rewiring, as the case we found in *NRAP* (Figure 6F), which could affect the myofibril assembly^[^ ^45^ ^]^ during SMT development.

## 4 Conclusion

In summary, we have constructed high‐resolution Hi‐C diploid maps, with a maximum resolution of 1.2 kb in skeletal muscle tissues from hybrid chickens over three developmental stages. Homolog pairing probably occurred in these hybrid chicken nuclei. Dynamic hierarchical chromatin architectures were closely linked to the transcriptional alterations taking place throughout the development and growth of skeletal muscle. Parent‐of‐origin‐specific chromatin conformation supported the lack of genomic imprinting identified in chickens. Although PEI differences between broiler and layer haplotypes were associated with genetic variation between breeds, only a limited number of breed‐specific variations are likely to contribute to phenotypic divergence in skeletal muscle potentially through allelic PEI rewiring, highlighting the remarkable redundancy in chromatin contacts within rewired enhancers carrying breed‐specific variants. Our haplotype‐resolved survey of 3D genome architecture in the F1 hybrid progeny greatly expands our understanding of how 3D chromatin organization affects the development and growth of chicken SMT through transcriptional programming, and provides candidate non‐coding variants in regulatory elements for functional validation of molecular drivers underlying phenotypic divergence between the broiler and layer chickens.

## 5 Experimental Section

### Ethics statement

The Institutional Animal Care and Use Committee in the College of Animal Science and Technology, Sichuan Agricultural University, Sichuan, China approved the animal maintenance and experimental procedures under permit No. 20220194. Ethical treatment was ensured and took special care to prevent animal suffering throughout the procedure.

### Animal Experiments and Sampling

To investigate the haplotype scale, reciprocal crosses were performed between two indigenous chicken breeds: Broiler (TB) and Layer (TL). This involved two forward crosses (TB♂×TL♀) and two reverse crosses (TL♂×TB♀) (Figure 1A). Female F1 hybrids at three distinct developmental stages, namely embryonic day 15 (E15; *n* = 4, including two hybrids from forward crosses and two from reverse crosses), hatching day (D1; *n* = 4), and 30 days after hatching (D30; *n* = 4), were sampled. Calf and thigh muscle tissues were collected from the hybrids and snap‐frozen in liquid nitrogen for subsequent in situ Hi‐C, RNA‐seq, and long‐read genome sequencing assays. Additionally, whole blood samples collected from the F0 parents (four broilers and four layers) and additional purebred individuals (eight broilers and eight layers), and fetal (E15 stage) or liver tissue samples (D1 and D30 stages) of the twelve F1 hybrids were obtained for genotyping via short‐read genome sequencing.

### Short‐Read Genome Sequencing and Short Variant Calling

Genomic DNA was extracted from whole blood samples (from 24 purebred individuals) and fetal or liver tissues (from twelve F1 hybrids) using the TIANamp Genomic DNA Kit (TIANGEN, DP304). Sequencing libraries were prepared using the genomic DNA and sequenced on the MGISEQ‐2000 platform (BGI Inc., Shenzhen, China) with paired‐end sequencing of 150 bp (PE150). High‐quality sequencing data (≈42.09× or 44.84 Gb per sample) were aligned to the chicken reference genome (GRCg6a, Ensembl release 104) using the Burrows‐Wheeler Aligner (BWA, v 0.7.8).^[^ ^53^ ^]^ Optical and PCR duplicates were eliminated using Picard MarkDuplicates (v 2.0.1). SNVs (single nucleotide variants) and short indels (insertions and deletions) were called using the Genome Analysis Toolkit (GATK, v 3.8) HaplotypeCaller.^[^ ^54^ ^]^ High‐confidence SNVs from all individuals were merged and subjected to principal component analysis (PCA) using GCTA (v 1.93.2).^[^ ^55^ ^]^ To maximize the number of variants for haplotype phasing, the variants of each family (including both parents and three F1 hybrids sampled at three stages) were combined using GVCFGenotyper.

### Reconstruction of haplotype‐resolved Hi‐C maps

Five in situ Hi‐C libraries were generated for each of the 12 F1 hybrid chicken skeletal muscle tissue (SMT) samples, following the previously described method^[^ ^50a^ ^]^ with minor modifications. Prior to mapping, heterozygous SNVs of F1 hybrids were replaced with ‘N’ bases in the chicken reference genome. Subsequently, the high‐quality Hi‐C reads were aligned to the variant‐masked genome. The standard pipeline of HiC‐Pro (v 2.9.0)^[^ ^56^ ^]^ was used to obtain valid Hi‐C contacts.

Chromosome‐level haplotypes (*n* = 24) were constructed for the F1 hybrids, utilizing both family genotypes and Hi‐C data of the F1 hybrids, as described previously (Figure S3, Supporting Information).^[^ ^57^ ^]^ Haplotype phasing of the valid Hi‐C contacts mapped on autosomes was performed using SNV phasing, local imputation, and HaploHiC software (v 0.32).^[^ ^58^ ^]^ This process was based on the number of informative SNVs in each read pair (Figure S2B,C, Supporting Information). 24 haplotype‐resolved intra‐chromosomal Hi‐C maps and 12 haplotype‐resolved inter‐chromosomal Hi‐C maps were generated using the phased Hi‐C reads. The maps were normalized using the Knight‐Ruiz (KR) algorithm^[^ ^59^ ^]^ and the quantile method.^[^ ^60^ ^]^ The similarity of normalized intra‐chromosomal contact matrices at 20‐kb resolution among haplotypes was evaluated using HiCRep,^[^ ^61^ ^]^ GenomeDISCO,^[^ ^62^ ^]^ and QuASR‐Rep^[^ ^63^ ^]^ with default parameters.

### Analysis of Haplotype‐Resolved Hi‐C Maps

The 3D genome architectures of hybrid chicken SMT were reconstructed using the normalized contact matrices from haplotype‐resolved Hi‐C data. This was achieved through the utilization of the miniMDS^[^ ^64^ ^]^ program. The resulting 3D structures were visualized using PYMOL (The PyMOL Molecular Graphics System, v 2.5.2 Schrödinger, LLC.). To quantify the intensity of colocalization between homologous chromosome pairs, the homolog pairing score (HPS) based on the normalized inter‐chromosomal Hi‐C maps were calculated at a resolution of 20 kb, following a previously described method.^[^ ^17^ ^]^ A/B compartments by performing PCA and using the A‐B index were identified, as described in previous studies,^[^ ^65^ ^]^ with haplotype‐resolved intra‐chromosomal contact matrices at resolutions of 20 and 100 kb. The similarity in compartmentalization among the haplotypes (*n* = 24) was assessed using Pearson's correlation coefficient (*r*) of the A‐B index. Differential compartments, including A/B switched and A/B variable compartments, were identified between haplotypes of different developmental stages, parents of origin, and parental breeds. TADs (topologically associating domains) at a resolution of 20 kb were determined using the Directionality Index (DI)^[^ ^65^, ^66^ ^]^ and the Insulation Index (IS),^[^ ^67^ ^]^ following established methods. The Measure of Concordance (MoC)^[^ ^68^ ^]^ and Variation of Information (VI)^[^ ^69^ ^]^ were employed to evaluate the similarity of TADs among haplotypes. TAD boundary shifts were identified by examining the changed boundary bins that exhibited significantly differential local boundary scores (LBS)^[^ ^70^ ^]^ (|ΔLBS| ≥0.2, *P* <0.05, paired Student's *t*‐test) between neighboring developmental stages, different parents of origin, and distinct parental breeds. Potential PEIs were identified using the PSYCHIC algorithm^[^ ^21^ ^]^ with default parameters. To investigate the regulatory effects of multiple enhancers on a gene, a regulatory potential score (RPS) was calculated for each gene using high‐confidence PEIs, as previously reported.^[^ ^23^ ^]^ Genes with differential RPS were identified between haplotypes of different developmental stages, parents of origin, and parental breeds.

### Allelic Transcriptome Profiling

Total RNA was extracted from the twelve SMT samples of F1 hybrids using the RNeasy Mini Kit (Qiagen), following the manufacturer's instructions. The integrity and concentration of the RNA were assessed using an Agilent 2100 Bioanalyzer and RNA 6000 Nano Kit (Agilent Technologies, Palo Alto, CA, USA). Twelve poly(A)‐ captured RNA‐seq libraries were constructed, one for each sample, following a standard protocol. Subsequently, the libraries were sequenced using the MGI DNBSEQ T7 platform with PE150 sequencing. The high‐quality RNA‐seq reads were aligned to the chicken reference genome using STAR (v 2.6.0c).^[^ ^71^ ^]^ The Kallisto software (v 0.44.0)^[^ ^72^ ^]^ was used to quantify the total expression level of genes (*n* = 19,328) was aligned. To assess allelic expression, Allelome.PRO^[^ ^13^ ^]^ was employed, utilizing the phased exonic SNVs. The similarities in allelic gene expression were estimated by calculating Spearman's correlation coefficients.

### Long‐Read Sequencing and Identification of Large Indels

Long‐read sequencing (Oxford Nanopore Technologies, ONT) was performed using genomic DNA extracted from the fetal or liver tissue of the twelve F1 hybrid chickens. For haplotype resolution of the long reads, short reads from the two parental haplotypes were used to assign the long reads into haplotype‐specific sets. This was achieved by identifying the presence of haplotype‐specific *k*‐mers using the trio binning method^[^ ^73^ ^]^ with the TrioCanu module of the Canu assembler (v 1.8). Subsequently, the haplotype‐resolved long reads were mapped to the chicken reference genome using NGMLR (v 0.2.7)^[^ ^74^ ^]^ with default parameters. Large indels with a length of 50 bp or greater were called using Sniffles (v 1.0.11)^[^ ^74^ ^]^ from the uniquely aligned reads. The identified indels were then merged using the software SURVIVOR.^[^ ^75^ ^]^

### Evaluating Sequence Divergence Between the Broilers and Layers

The sequence divergence between broiler and layer haplotypes were quantitatively assessed by calculating the identity score (IDS). The IDS represents the percentage of identical nucleotides in a given genomic region, considering all SNVs, short and large indels. Additionally, to evaluate the sequence divergence at the population level, the *F*‐statistics (*F*_ST_) between the two breeds were estimated. Genotype data from 24 purebred individuals and vcftools (v 0.1.15)^[^ ^76^ ^]^ were utilized to analyze phased SNVs and short indels identified in the F1 hybrids. For the large indels identified in the 24 haplotypes of F1 hybrids, the genotype in each purebred individual was first inferred using the paragraph software (v 2.4a),^[^ ^77^ ^]^ followed by *F*_ST_ calculation using vcftools.

### Functional enrichment analysis

Functional enrichment analysis was conducted using the Metascape^[^ ^78^ ^]^ tool with default parameters. The input genes were converted to their human orthologs. The target species was set as *Homo sapiens* (human), and the background set comprised all annotated genes. The Gene Ontology‐biological processes (GO‐BP) and KEGG pathways were used as the test sets. Statistically significant terms were considered as outputs.

### Statistical Analysis

Statistical significance was assessed using the Student's *t*‐test, Wilcoxon rank‐sum test, or Fisher's exact test in R. The data presented are as mean ± standard deviation (SD) or specifically indicated in figure legends. For further details, refer to the Supplementary Methods.

## Conflict of Interest

The authors declare no conflict of interest.

## Author Contributions

J.L., Y.L., and D.L. contributed equally to this work. M.L. and Y.L. designed the experiments. D.L., M.H., H.K., and T.W. performed animal work and prepared biological samples. J.L. performed sequencing and designed the bioinformatics analyses. Y.L., Z.C., and Y.G. performed the data analysis. J.B. and J.Z. performed 3D genome structure reconstruction. L.J., Q.T., and F.K. contributed to the interpretation of data. J.L. and Y.L. drafted the paper. M.L. revised the paper. J.L., M.L., and L.J. acquired the funding.

## Supporting information

Supporting Information

## Data Availability

All sequencing data produced in this study were submitted to the Genome Sequence Archive of China National Center for Bioinformation (GSA; https://ngdc.cncb.ac.cn/gsa) under the accession number CRA009726.
