# Supplementary material for: Building Haplotype‐Resolved 3D Genome Maps of Chicken Skeletal Muscle
Source: Adv Sci (Weinh). 2024 Apr 6;11(24):2305706. doi: 10.1002/advs.202305706 (PMC11200017; doi:10.1002/advs.202305706)
Supplement: Supplementary file 1 — Supporting Information [file ADVS-11-2305706-s001.pdf]

ADVANCED SCIENCE
Open Access

Supporting Information

for *Adv. Sci.*, DOI 10.1002/advs.202305706

Building Haplotype-Resolved 3D Genome Maps of Chicken Skeletal Muscle

Jing Li, Yu Lin, Diyan Li, Mengnan He, Hua Kui, Jingyi Bai, Ziyu Chen, Yuwei Gou, Jiaman Zhang, Tao Wang, Qianzi Tang, Fanli Kong, Long Jin and Mingzhou Li**

Supporting Information

Building Haplotype-resolved 3D Genome Maps of Chicken Skeletal Muscle

Jing Li, Yu Lin, Diyan Li, Mengnan He, Hua Kui, Jingyi Bai, Ziyu Chen, Yuwei Gou, Jiaman Zhang, Tao Wang, Qianzi Tang, Fanli Kong, Long Jin, and Mingzhou Li**

Table of Contents

Supplementary Figures 1–21

Figure S1. Summary of the whole-genome sequencing data for the two families of forward crosses and the two families of reverse crosses (n = 20 individuals).

Figure S2. Phasing Hi-C data for the 12 F1 hybrid samples.

Figure S3. Construction of haplotypes using trio-based genomic and Hi-C data.

Figure S4. Summary of RNA-seq data for the 12 F1 hybrid samples.

Figure S5. Characteristics of spatial organization of diploid chicken genomes in the skeletal muscle tissue.

Figure S6. Basic sequence feature and spatial location of each chromosome in the chicken genome.

Figure S7. Circos plot displaying the global distribution of sequence features, 3D architecture traits, and gene expression profiles in F1 hybrid chickens.

Figure S8. Basic characteristics of homolog pairing in the hybrid chicken samples.

Figure S9. Basic features of haplotype-resolved compartments in the hybrid chicken samples.

Figure S10. Basic features of haplotype-resolved TADs in the hybrid chicken samples.

Figure S11. Basic features and expression regulation of haplotype-resolved PEIs in the hybrid chicken samples.

Figure S12. Similarity in haplotype-resolved chromatin architecture and gene expression for hybrid chicken skeletal muscle samples.

Figure S13. Outputs of functional enrichment analysis for genes located in stage-restricted active compartments in comparison to neighboring stages.

Figure S14. A-B index heatmaps of two sets of genes involved in myogenesis and skeletal muscle development.

Figure S15. Outputs of functional enrichment analysis for the differential RPS genes between neighboring stages.

Figure S16. PEI rewiring of nine representative genes with differential RPS between neighboring stages.

Figure S17. Evaluation of differences in chromatin hierarchical structures between maternal and paternal haplotypes for the 106 chicken orthologs of empirical imprinted genes.

Figure S18. Characteristics of differential compartments between parental breeds in the hybrid chickens.

Figure S19. PEI rewiring of representative genes with differential RPS between breeds.

Figure S20. Effects of genomic variants between breeds on PEI wiring in the hybrid chickens.

Figure S21. Detection of homolog pairing with heterozygous SNVs at different confidence levels to assign parental origins of chromatin interactions.

Supplementary Methods

1. **Identification of SNVs and short InDels**
2. ***In situ* Hi-C library sequencing**
3. **Reconstruction of haplotype-resolved Hi-C maps**
    - 3.1. Hi-C data mapping
    - 3.2. Building chromosome-level haplotypes
    - 3.3. Constructing haplotype-resolved Hi-C maps
4. **Analysis of haplotype-resolved Hi-C maps**
    - 4.1. Modelling of diploid 3D genome structures
    - 4.2. Calculation of homolog pairing score (HPS)
    - 4.3. Identification of A/B compartments
    - 4.4. Detecting variable compartments between haplotypes
    - 4.5. Calling of topologically associating domains (TADs)

4.6. Detection of TAD boundary shifts

4.7. Identification of promoter-enhancer interactions (PEIs)

4.8. Detection of differential RPS genes

5. Quantitation of allelic gene expression

6. Long-read genome sequencing

7. Identification of large insertions and deletions (InDels)

8. Effects of genetic variants on PEIs

8.1. Calculation of the identity score (IDS)

8.2. Calculation of *F*-statistics (F_{ST})

Supplementary Figures

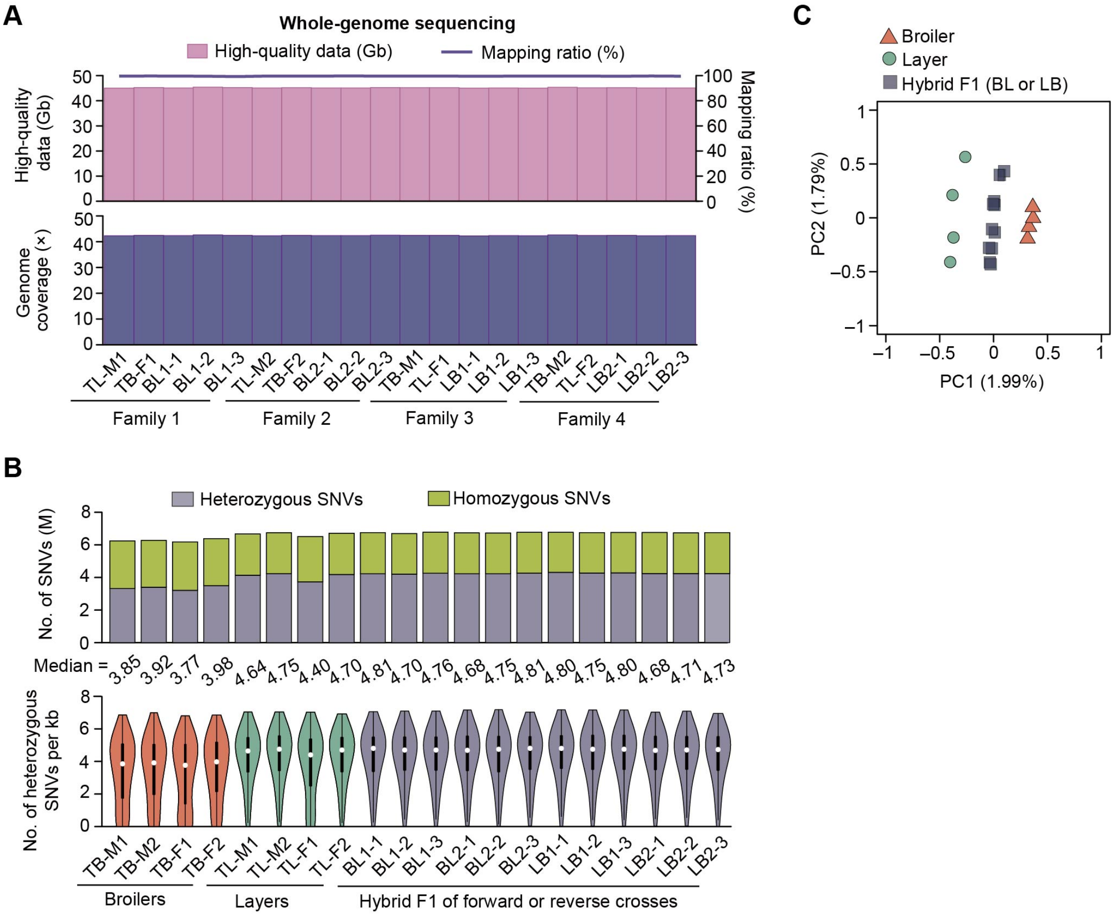

Figure S1. Summary of the whole-genome sequencing data for the two families of forward crosses and the two families of reverse crosses (*n* = 20 individuals). A) Volume and coverage of high-quality whole-genome sequencing data for four families, with two parents and three hybrids (one hybrid per time point) per family. B) Genomic SNV number and heterozygous SNV frequency in each individual (*n* = 20). C) Principal component analysis (PCA) of all individuals (*n* = 20) constructed using genomic SNVs.

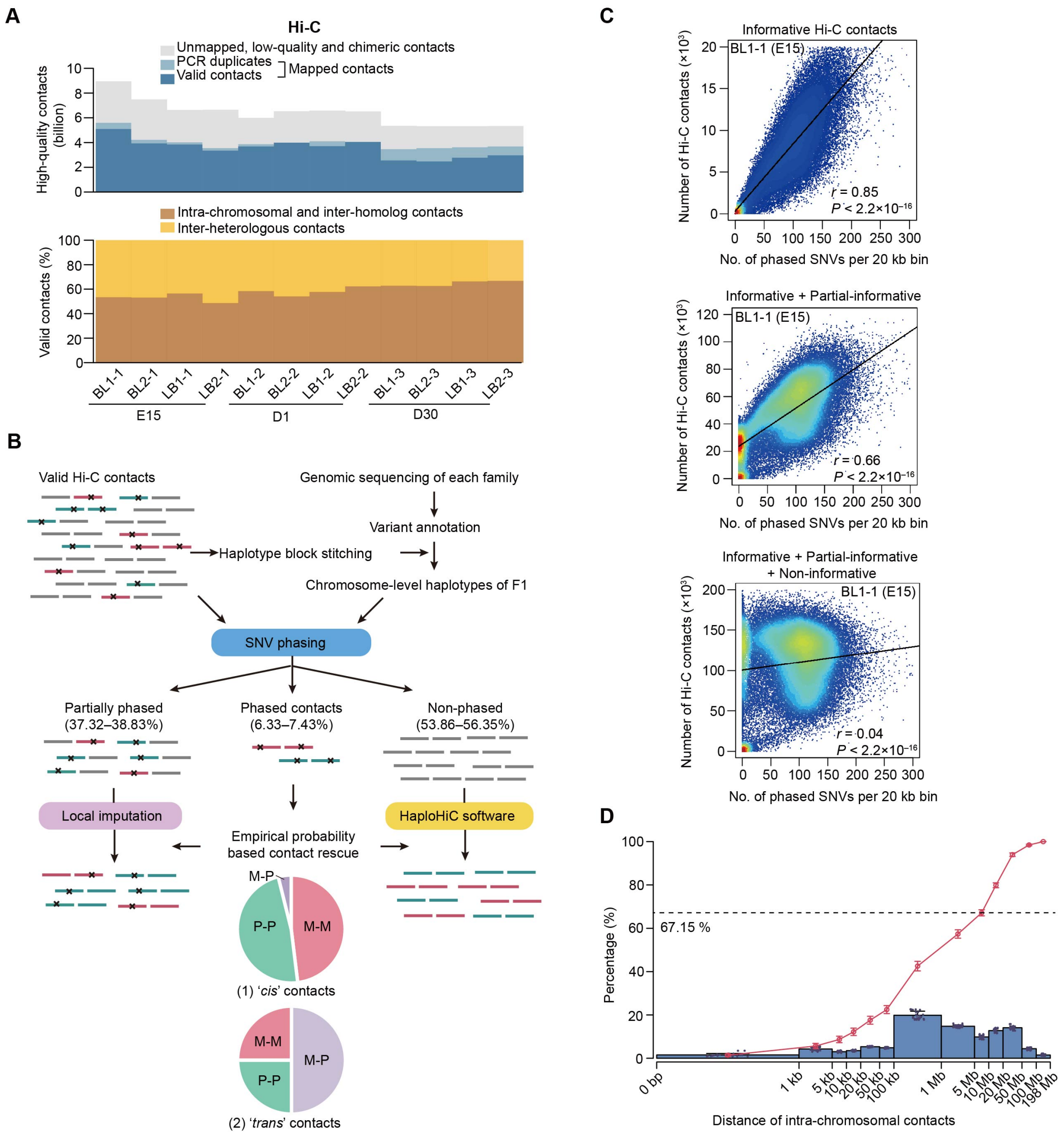

Figure S2. Phasing Hi-C data for the 12 F1 hybrid samples. A) Summary of *in situ* Hi-C data of the skeletal muscle tissue in hybrid chickens sampled at three developmental stages. Number of valid contacts and proportions of unphased intra- ('*cis*') and inter-chromosomal ('*trans*') contacts are shown for each sample. B) Workflow of Hi-C contact phasing to construct haplotype-resolved Hi-C maps using the SNV phasing method, local imputation approach, and HaploHiC[1] algorithm. After SNV phasing, partially phased and non-phased contacts can be further assigned to their parental origins using the local imputation method and HaploHiC respectively, based on the empirical probabilities of various kinds of broadly defined '*cis'* (including intra-maternal [denoted as 'M-M'], intra-paternal ['P-P'] and inter-homologous ['M-P']) or '*trans'* (including inter-maternal [denoted as 'M-M'], inter-paternal ['P-P'] and inter-heterozygous ['M-P']) phased contacts. C) Density plots showing the correlation between the number of phased SNVs and the count of phased informative contacts only (upper), phased informative and partial informative contacts (middle), and all phased (informative, partial informative, and non-informative) contacts (lower), respectively. Allele assignment of all categories of Hi-C contacts can efficiently eliminate the negative effects caused by differences in SNV density across the

genome. Spearman's correlation coefficients are indicated on the plots. Data of a F1 hybrid chicken skeletal muscle sample (BL1-1) at E15 stage is shown. D) Insert size distribution of haplotype-resolved intra-chromosomal Hi-C contacts. The column represents the proportion of contacts within each distance interval. The dot on the column indicates the percentage of each sample. The red curve shows the cumulative percentage. Data is represented as means ± SD of the 24 haplotypes. On average, 67.15% of the intra-chromosomal contacts (indicated with the dashed lines) occurred mainly within 10 Mb.

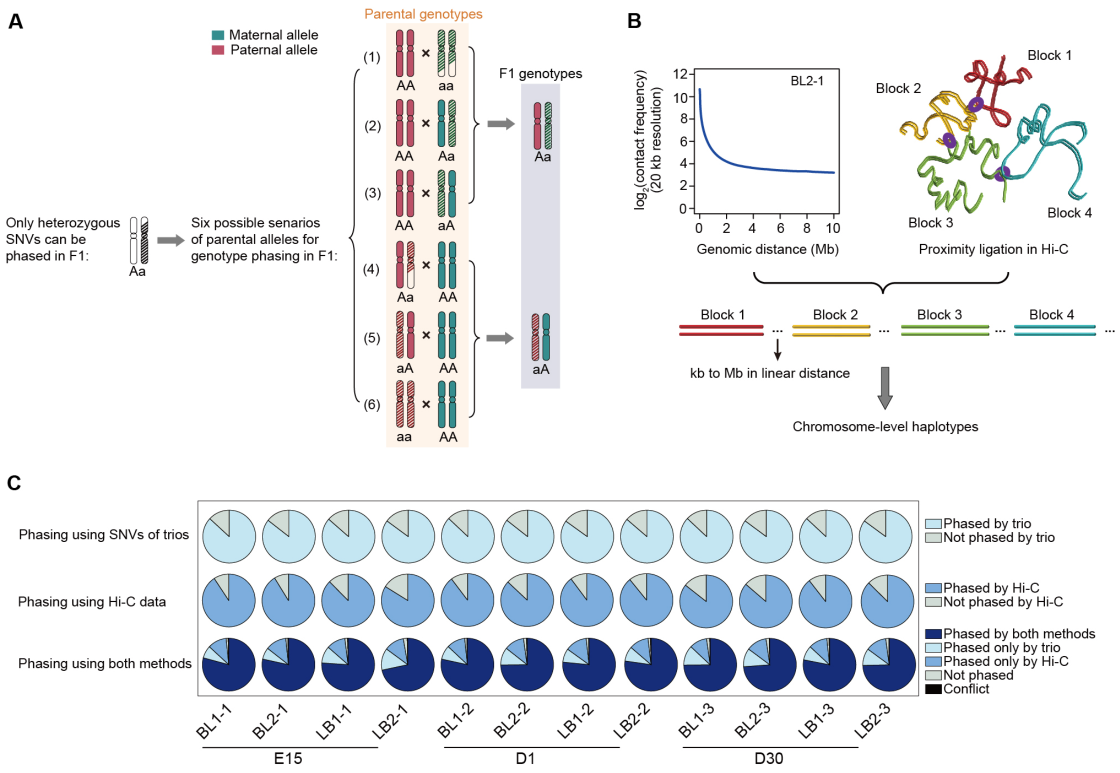

Figure S3. Construction of haplotypes using trio-based genomic and Hi-C data. A) Illustration of SNV phasing based on the genotypes of both parents and hybrid offspring. For a certain heterozygous locus in hybrid chicken, the heterozygous locus can be successfully phased in hybrids when at least one parent is homozygous. B) Illustration of haplotype construction using Hi-C contacts and the HapCUT2 algorithm.[2] Upper left: distance-dependent interaction decay, drawn using Hi-C contacts at 20-kb resolution. Upper right: proximity ligation of different haplotype blocks in Hi-C. Lower: elongation of haplotype blocks based on the distance-dependent decay feature of Hi-C contacts. C) SNV phasing efficiency using different methods, including genotyping of parent and offspring chickens, Hi-C contacts of hybrid chickens, and the combination of both these methods.

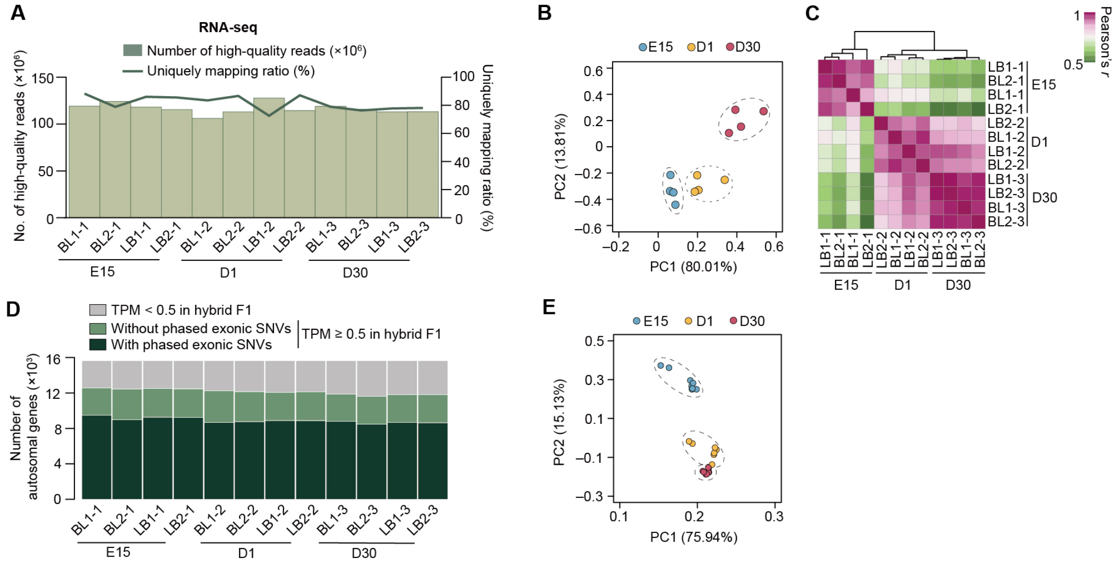

Figure S4. Summary of RNA-seq data for the 12 F1 hybrid samples. A) Volume and mapping ratio of high-quality poly(A) captured RNA-seq data. B) PCA of gene expression profiles using the unphased RNA-seq data, showing a developmental stage-dominant pattern. C) Spearman's *r* heatmap of gene expression profiles using the unphased RNA-seq data, showing a developmental stage-dominant pattern. D) Number of autosomal genes with haplotype-resolved expression data in the 12 hybrid chicken samples. E) PCA of haplotype-resolved gene expression profiles using phased RNA-seq data, showing a developmental stage-dominant pattern.

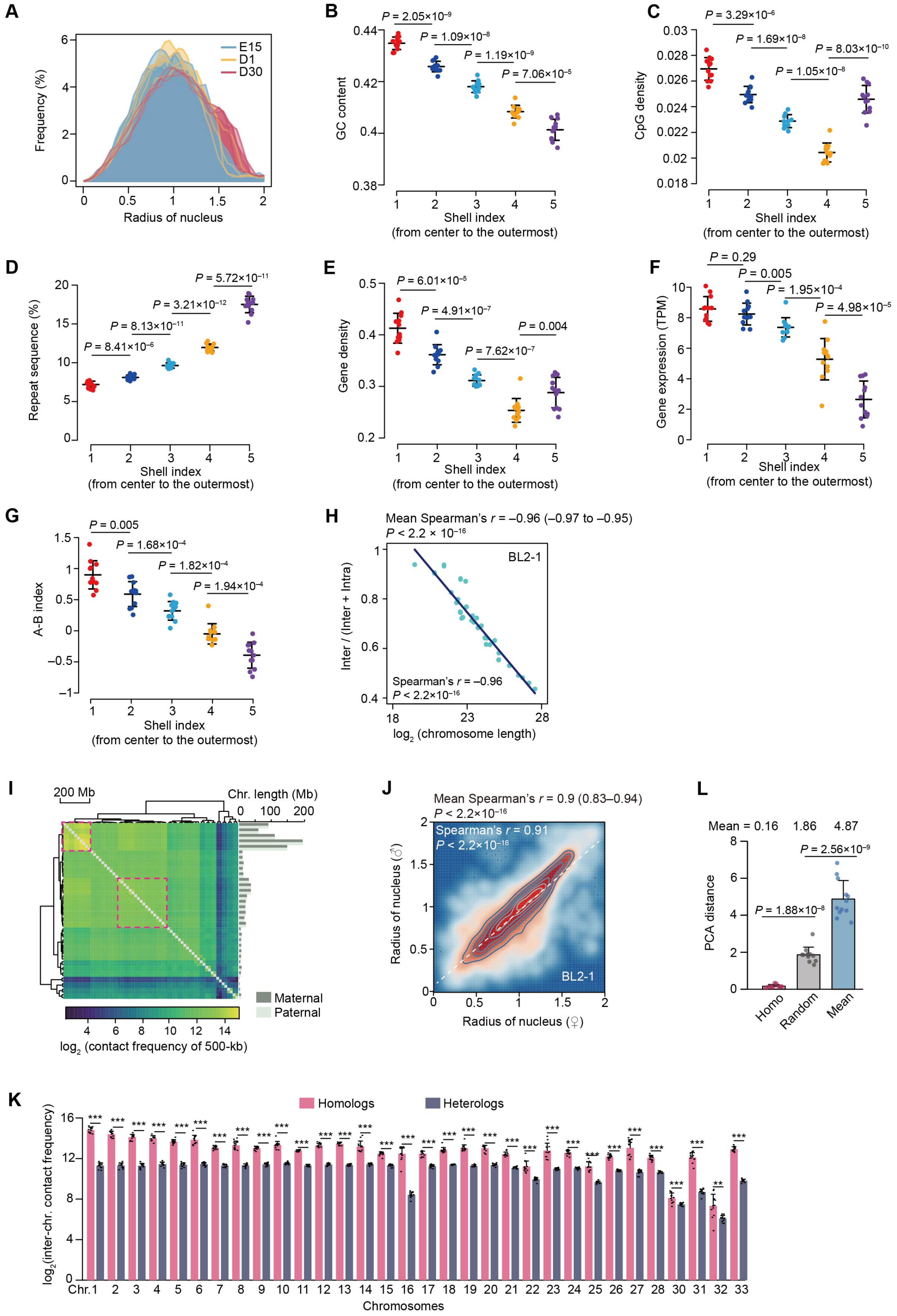

Figure S5. Characteristics of spatial organization of diploid chicken genomes in the skeletal muscle tissue. A) Frequency distribution of 20-kb genomic bins along the radius of the nucleus across the 12 hybrid chicken samples. Nucleotides were concentrated in the nucleus interior but depleted in the nucleolus and the outermost part of the nucleus. B–G) Correlation between sequence feature and spatial position in the nucleus, including GC

content (B), CpG density (C), percentage of repeat sequence (D), gene density (E), expression level (F), and A-B index (G). Sequence features and spatial positions were compared between five nuclear shells with equal nucleotides in each shell (from center to the outermost were indexed as 1 to 5). The dots represent values of each sample (n = 12). The P values were calculated using the paired Student's t-test. It demonstrates that GC-rich, gene rich, transcript-abundant chromatins were more likely to inhabit the interior of the nucleus; whereas GC-poor, transcript-sparse chromatins and repeat sequences were preferentially located in the nuclear periphery. H) Ratio of inter-chromosomal contacts to all Hi-C contacts (intra- and inter-chromosomal contacts) was negatively correlated with the chromosome length. I) Heatmap of inter-chromosomal Hi-C contacts showing chromosome territories (indicated by the dotted boxes in magenta) formed by chromosomes of similar lengths. J) Spatial organization of the two chromosomes of a homolog pair was highly correlated with each other. K) Inter-chromosomal contact frequencies between homologous chromosomes compared with the frequencies between heterologous chromosomes. The dots denote values of each sample (n = 12). The P values were calculated using a paired Student's t-test. ** $0.001 < P < 0.01$; *** $P < 0.001$. L) PCA distance between homologous chromosomes ('Homo') compared with that of randomly selected chromosomes ('Random') and mean values of all chromosome pairs ('Mean'). P values are from Student's t-test. The data of a hybrid chicken sample (BL2-1) at E15 stage is shown. In H–K and I, data of a hybrid chicken sample (BL2-1) at E15 stage is shown. Average Spearman's correlation coefficients of all samples are shown above the corresponding plots.

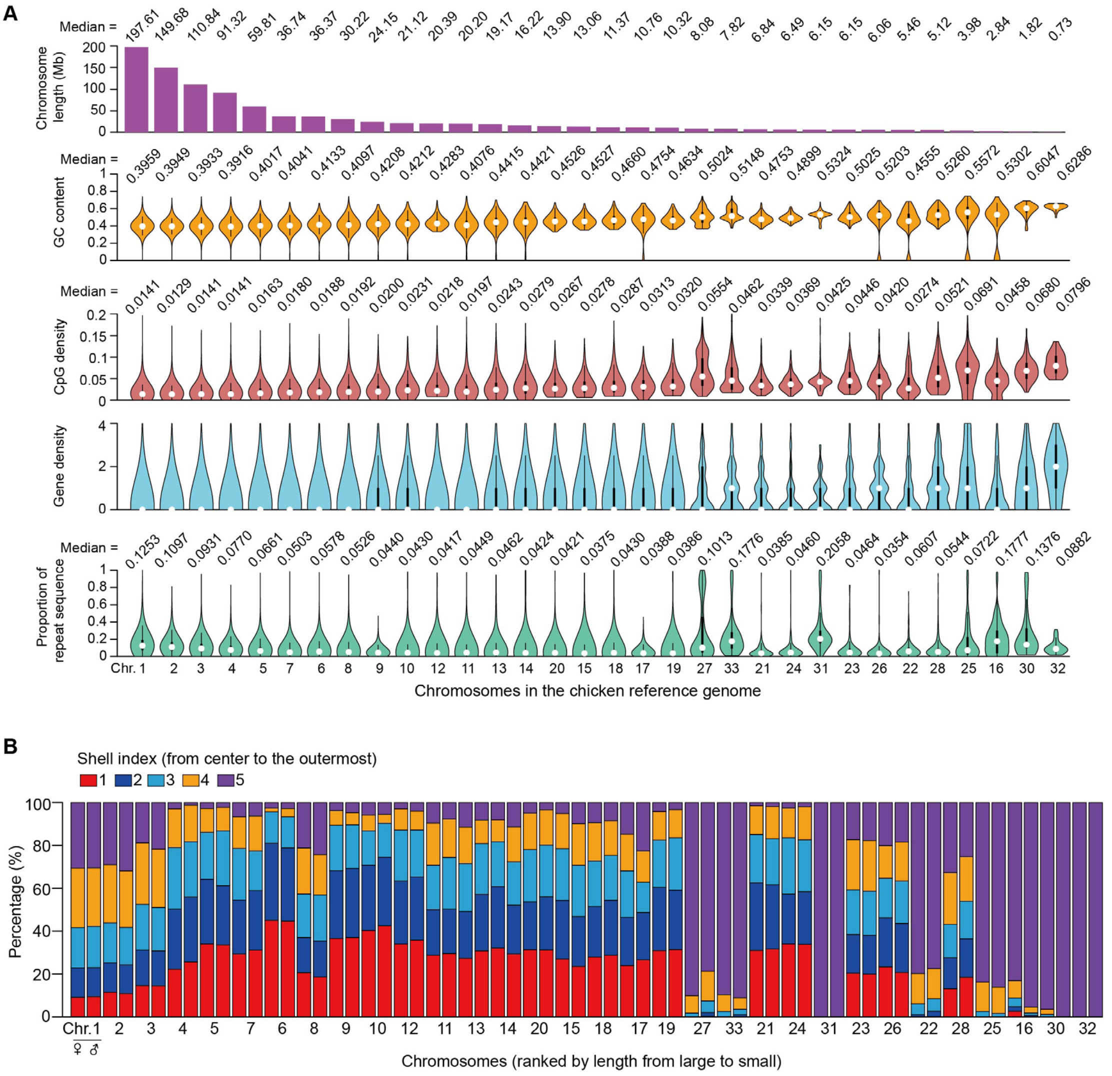

Figure S6. Basic sequence feature and spatial location of each chromosome in the chicken genome. A) Basic sequence feature of each autosome in the chicken reference genome (GRCg6a, Ensembl release 104). We partitioned each chromosome into contiguous 20 kb windows and subsequently calculated GC content, CpG density, gene density, and repeat element density for each window. The values of all windows on each chromosome were then used to generate these violin plots. B) Proportion of 20 kb genomic bins located in different nuclear shells in each autosome. The nucleus was divided into five equally spaced shells from the center to the outermost. Mean values of 12 hybrid chicken samples are shown.

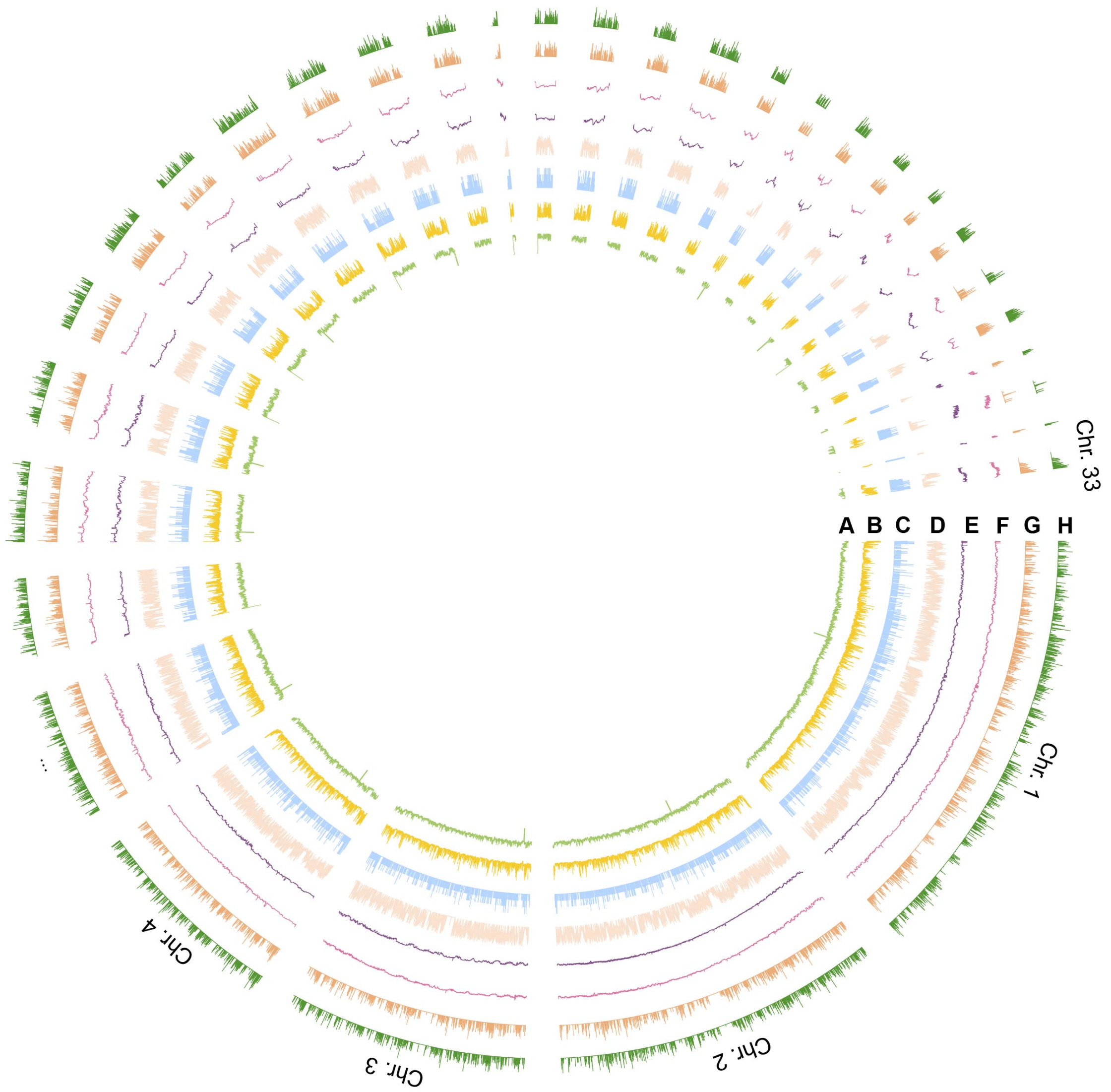

Figure S7. Circos plot displaying the global distribution of sequence features, 3D architecture traits, and gene expression profiles in F1 hybrid chickens. A) GC content. B) CpG frequency. C) Gene density of the chicken reference genome. D) Heterozygous SNV frequency. E,F) Nuclear radius of the maternal and paternal haplotype. G,H) TPM of the maternal and paternal haplotype. Data of a F1 hybrid chicken skeletal muscle sample (BL2-1) at E15 stage is shown.

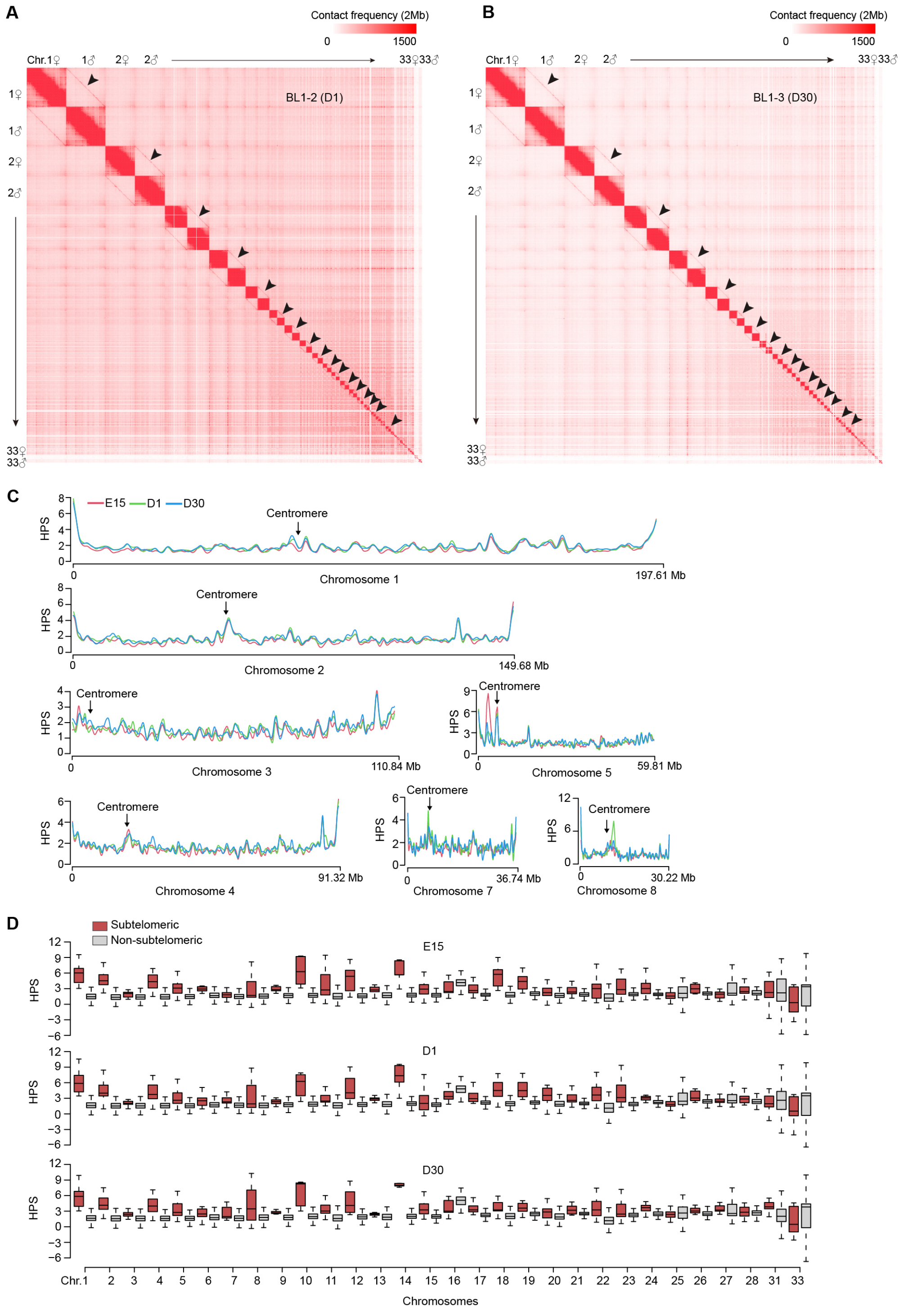

Figure S8. Basic characteristics of homolog pairing in the hybrid chicken samples. A,B) Close spatial localization of homologous chromosome pairs observed in a sample at D1 (A) and D30 (B), respectively, are indicated by the diagonal lines (black arrows) beside the intra-chromosomal interaction squares. Relatively intense chromatin interactions between homologs are shown on the Hi-C map. C) Homolog pairing levels along the chromosomes with determined centromeres. Centromeric regions and both ends (usually telomeres) of the chromosomes had high homolog pairing levels. D) Comparison of homolog pairing levels between subtelomeric (within 500 kb regions of telomeres) and non-subtelomeric regions. Subtelomeric regions are shown to have had more intense homolog pairing than other regions.

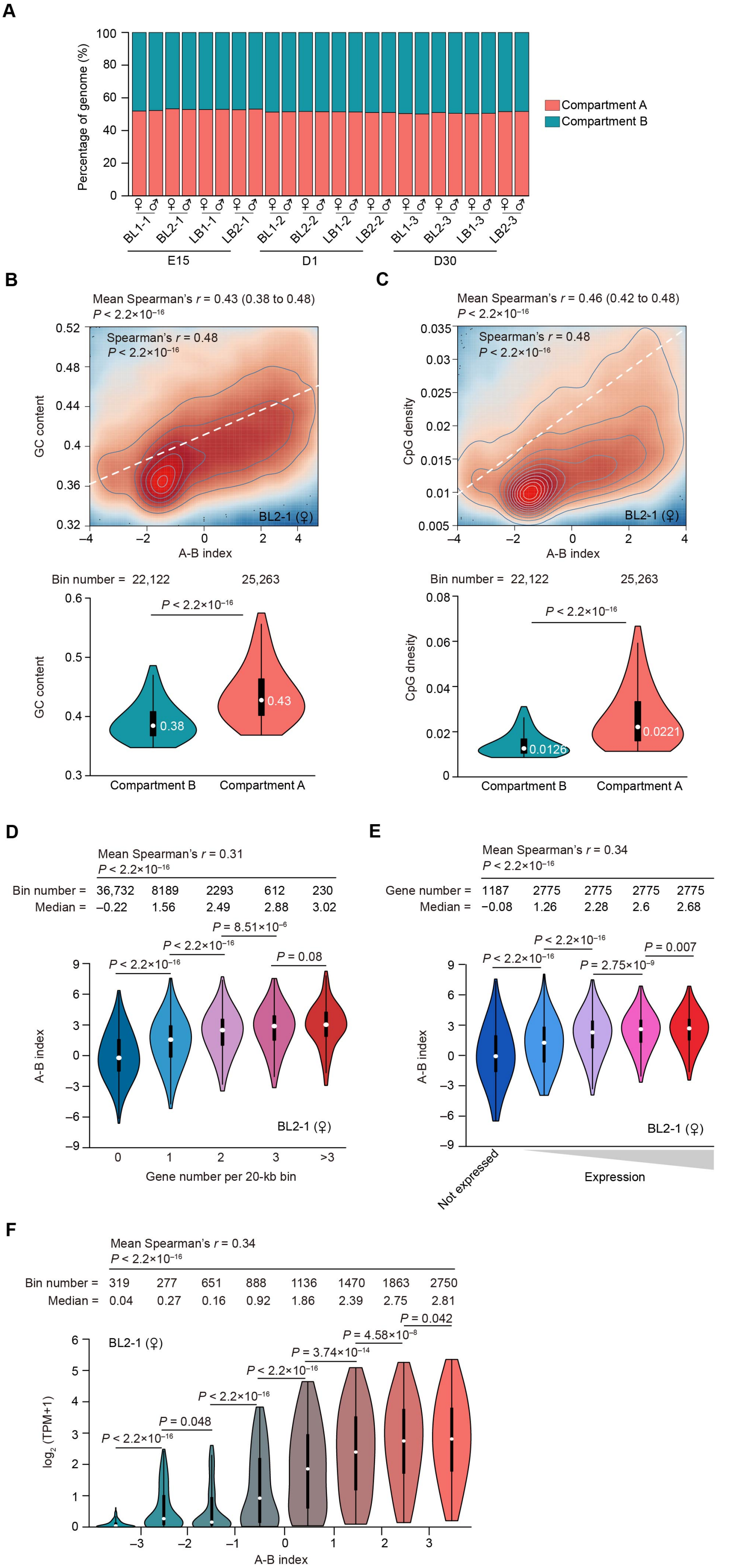
A
Percentage of genome (%)
Compartment A
Compartment B
BL1-1
BL2-1
LB1-1
LB2-1
BL1-2
BL2-2
LB1-2
LB2-2
BL1-3
BL2-3
LB1-3
LB2-3
E15
D1
D30
B
Mean Spearman's r = 0.43 (0.38 to 0.48)
P < 2.2×10^−16
Spearman's r = 0.48
P < 2.2×10^−16
GC content
A-B index
BL2-1 (♀)
Bin number = 22,122 25,263
P < 2.2×10^−16
0.38
0.43
Compartment B
Compartment A
C
Mean Spearman's r = 0.46 (0.42 to 0.48)
P < 2.2×10^−16
Spearman's r = 0.48
P < 2.2×10^−16
CpG density
A-B index
BL2-1 (♀)
Bin number = 22,122 25,263
P < 2.2×10^−16
CpG dnesity
0.0126
0.0221
Compartment B
Compartment A
D
Mean Spearman's r = 0.31
P < 2.2×10^−16
Bin number = 36,732 8189 2293 612 230
Median = −0.22 1.56 2.49 2.88 3.02
P = 8.51×10^−6
P < 2.2×10^−16
P < 2.2×10^−16
P = 0.08
A-B index
BL2-1 (♀)
0 1 2 3 >3
Gene number per 20-kb bin
E
Mean Spearman's r = 0.34
P < 2.2×10^−16
Gene number = 1187 2775 2775 2775 2775
Median = −0.08 1.26 2.28 2.6 2.68
P < 2.2×10^−16
P < 2.2×10^−16
P = 0.007
P = 2.75×10^−9
A-B index
BL2-1 (♀)
Not expressed
Expression
F
Mean Spearman's r = 0.34
P < 2.2×10^−16
Bin number = 319 277 651 888 1136 1470 1863 2750
Median = 0.04 0.27 0.16 0.92 1.86 2.39 2.75 2.81
P = 0.042
P = 4.58×10^−8
P = 3.74×10^−14
P < 2.2×10^−16
P < 2.2×10^−16
P = 0.048
P < 2.2×10^−16
BL2-1 (♀)
log2 (TPM+1)
A-B index

Figure S9. Basic features of haplotype-resolved compartments in the hybrid chicken samples. A) Length proportions of A/B compartments in the haploid genome (*n* = 24) of hybrid chicken skeletal muscle samples. B,C) Correlation between GC content (B) or CpG density (C) and A/B compartments. Upper: density plots demonstrate positive correlation between GC content (B) or CpG density (C) and A-B index (i.e., chromatin activity). Lower: comparison of GC content (B; 0.43 vs. 0.38, $P < 2.2\times10^{-16}$, Wilcoxon rank sum test) or CpG density (C; 0.0126 vs. 0.0221, $P < 2.2\times10^{-16}$, Wilcoxon rank sum test) between A and B compartments. D–F) Gene density and expression of A/B compartments with different activities. A-B index was moderately correlated with gene density (D; mean Spearman's *r* = 0.32 for the 24 haplotypes, ranging from 0.31 to 0.35) and expression level (E,F; mean Spearman's *r* = 0.39 for the 24 haplotypes, ranging from 0.31 to 0.49). A hybrid chicken sample at E15 stage is shown. *P* values were calculated using the Wilcoxon rank-sum test.

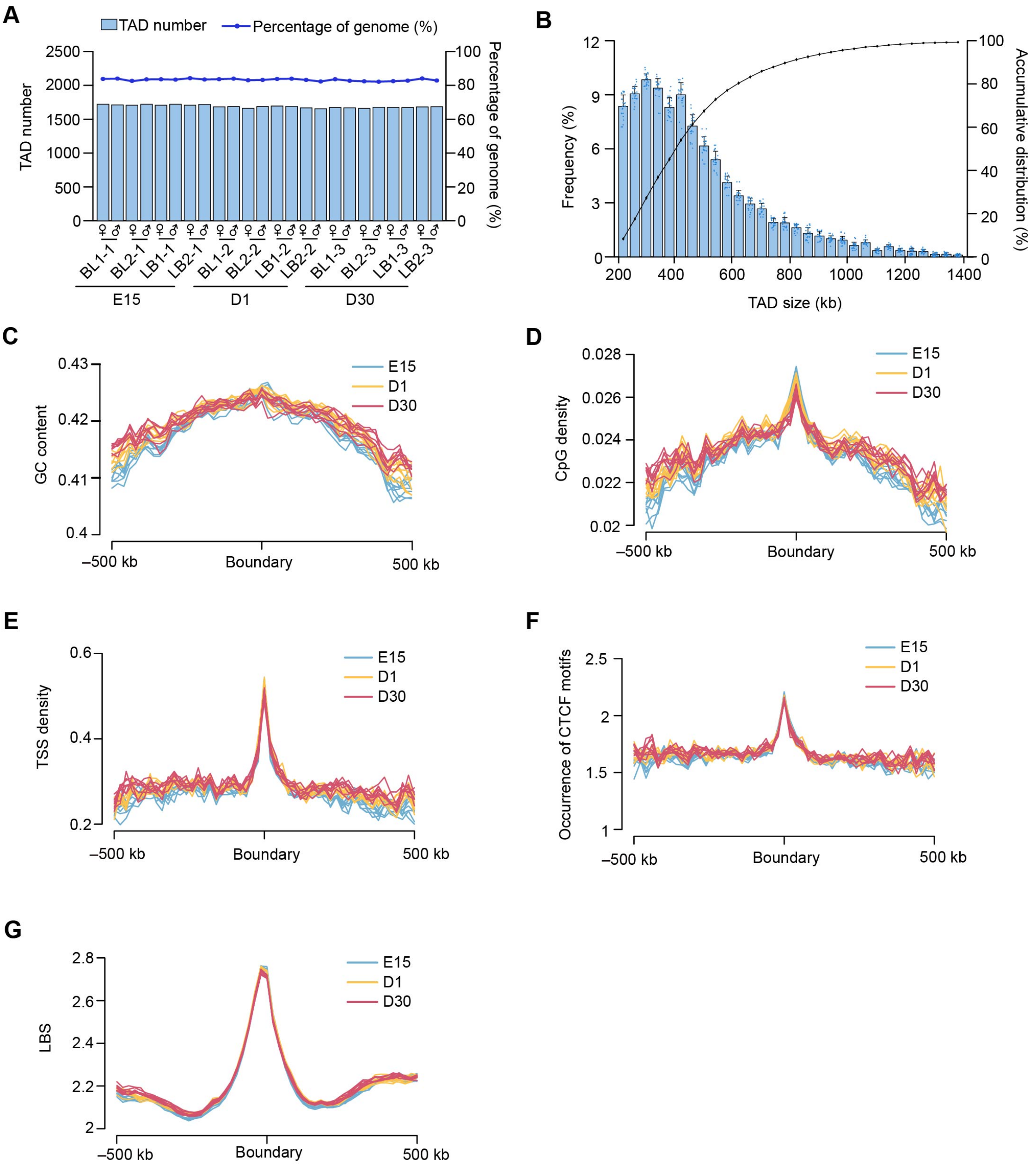

Figure S10. Basic features of haplotype-resolved TADs in the hybrid chicken samples. A) Numbers and genome coverage of TADs identified in the 24 haploid genomes of hybrid chicken samples. B) Size distribution of TADs identified in the haploid genomes of hybrid chicken samples. Data is shown as means ± SD. The dots represent haplotypes (*n* = 24). C–G) Qualification of GC content (C), CpG density (D), transcription start site (TSS) density (E), CTCF motif (F), and local boundary scores (LBS; G) across the up- and downstream 500 kb of TAD boundaries. TAD boundary was enriched for GC content, CpG islands, protein coding genes, and CTCF motifs as expected. TAD boundary also showed much higher LBS than flanking sequences, indicating much lower local contact frequency.

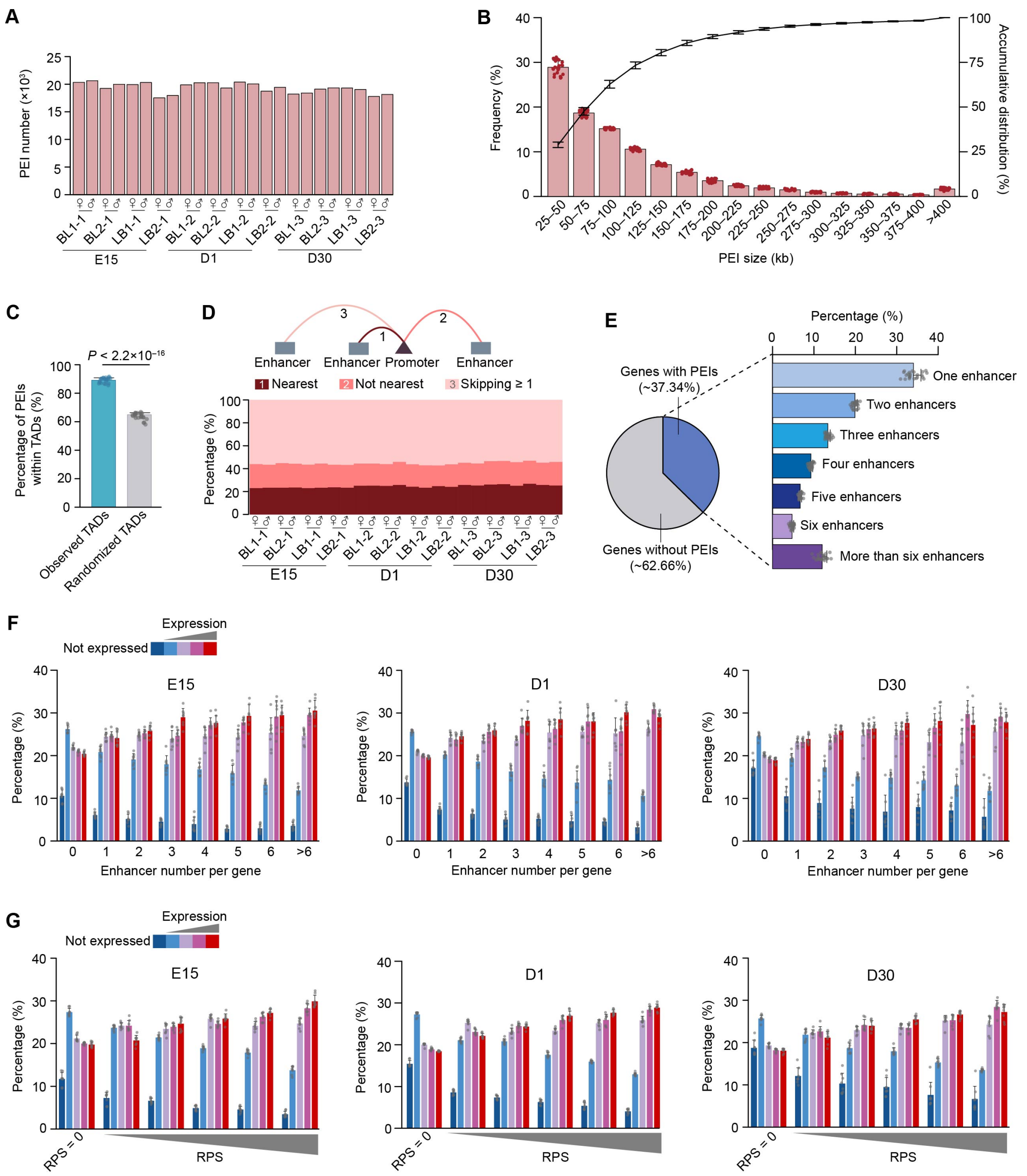

Figure S11. Basic features and expression regulation of haplotype-resolved PEIs in the hybrid chicken samples. A) Number of PEIs (19,310 on average) identified in the 24 haplotypes. B) Size distribution of PEIs. C) Percentage of PEIs located within TADs, which was significantly higher than the random situation ($P < 2.2\times10^{-16}$, paired Student's *t*-test). D) Percentage of protein coding genes interacting with the nearest, not nearest or skipping one or more enhancers. E) Percentage distribution of protein coding genes with different numbers of enhancers. F) Proportion of genes from each expression category interacting with zero to more than six enhancers, indicating that genes with more enhancers generally have higher expression. G) Proportion of genes from each expression category with different levels of RPS, indicating genes with larger RPS generally have higher expression. Genes with RPS > 0 were divided equally into five different percentiles. In B, C, and E–G, data is presented as means ± SD, and the dots represent the haplotypes (n = 24).

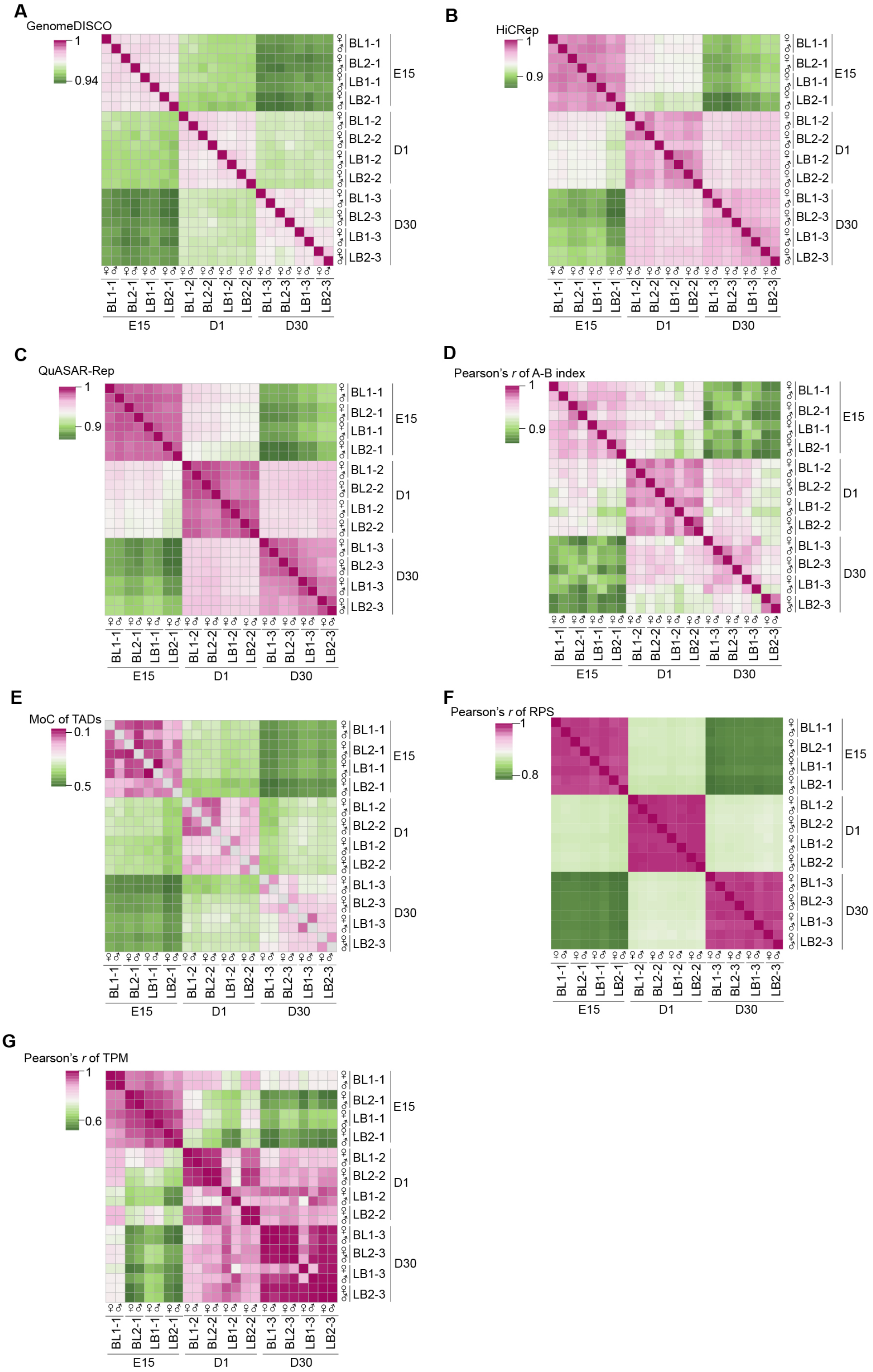

Figure S12. Similarity in haplotype-resolved chromatin architecture and gene expression for hybrid chicken skeletal muscle samples. A–C) Heatmaps of HiCRep, GenomeDISCO, and QuASAR-Rep among the haplotypes (n = 24) to evaluate similarity in the whole Hi-C map. D–F) Heatmaps of Pearson's correlation coefficients of A-B index, MoC of TADs, and regulatory potential score (RPS) of genes interacting with enhancers among haplotypes (n = 24) to evaluate the similarity in the compartmentalization, TAD organization, and PEI wiring. G) Heatmaps of Pearson's correlation coefficients of TPM among the haplotypes (n = 24) to evaluate similarity in gene expression profiles.

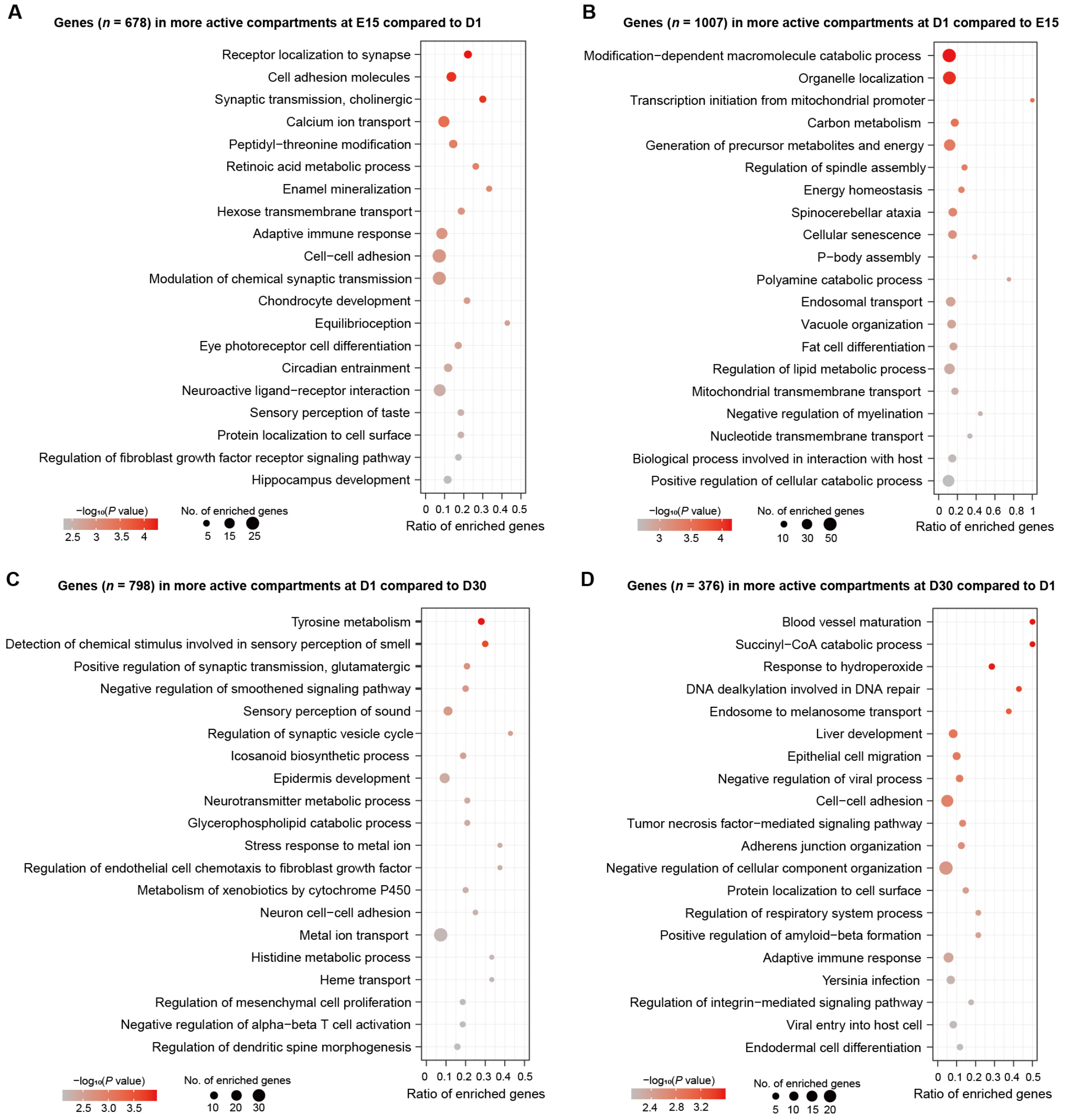

Figure S13. Outputs of functional enrichment analysis for genes located in stage-restricted active compartments in comparison to neighboring stages. A) Functional enrichment of genes located in more active compartments at E15 compared to D1 stage. B) Functional enrichment of genes located in more active compartments at D1 compared to E15 stage. C) Functional enrichment of genes located in more active compartments at D1 compared to D30 stage. D) Functional enrichment of genes located in more active compartments at D30 compared to D1 stage. The top 20 enriched terms are shown.

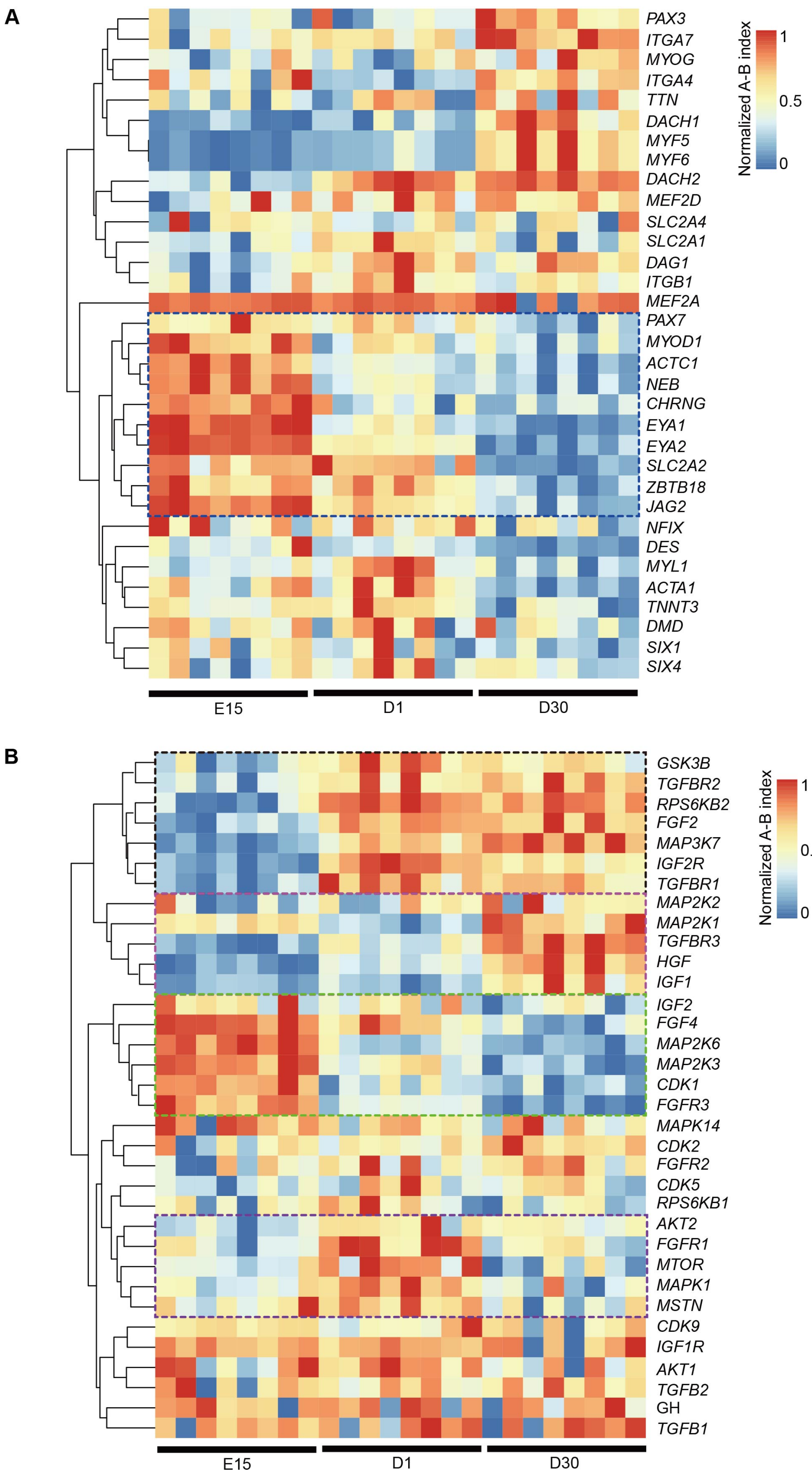

Figure S14. A-B index heatmaps of two sets of genes involved in myogenesis and skeletal muscle development. A) Heatmaps of A-B index for the markers (*n* = 33) that highly express in primary and intermediate myocytes during embryonic/fetal myogenesis.[3] The blue dotted box indicates the markers shifting from active compartmental status at E15 to repressive at D30. B) Heatmaps of A-B index for the genes (*n* = 34) encoding protein kinases and growth factors as well as growth factor receptors that are crucial for muscle development and growth in farm animals.[4] Colorful dotted squares indicate the genes with stage-dependent compartmentalization patterns.

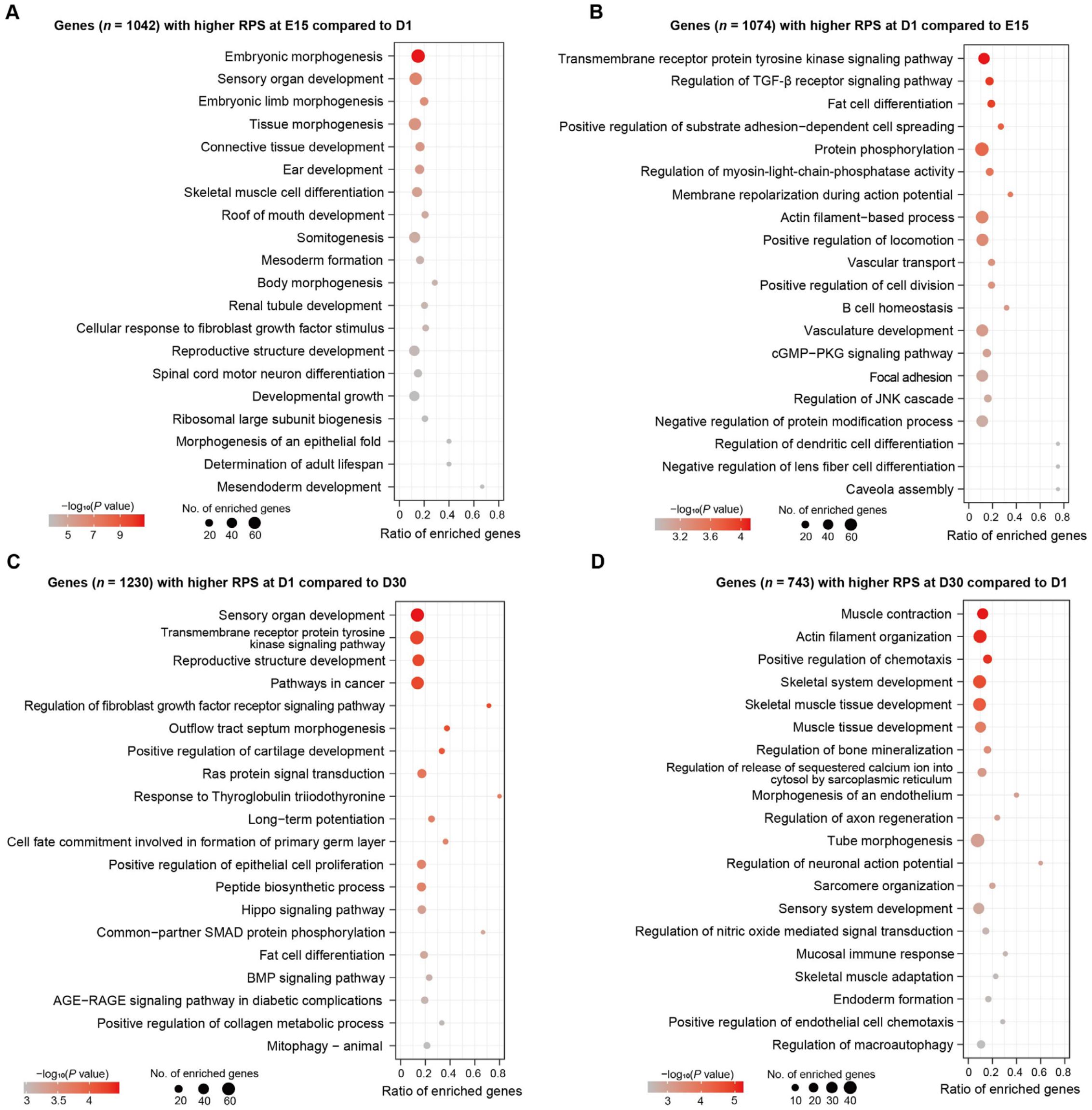

Figure S15. Outputs of functional enrichment analysis for the differential RPS genes between neighboring stages. A) Functional enrichment of genes with higher RPS at E15 compared to D1. B) Functional enrichment of genes with higher RPS at D1 compared to E15. C) Functional enrichment of genes with higher RPS at D1 compared to D30. D) Functional enrichment of genes with higher RPS at D30 compared to D1. The top 20 enriched terms are shown.

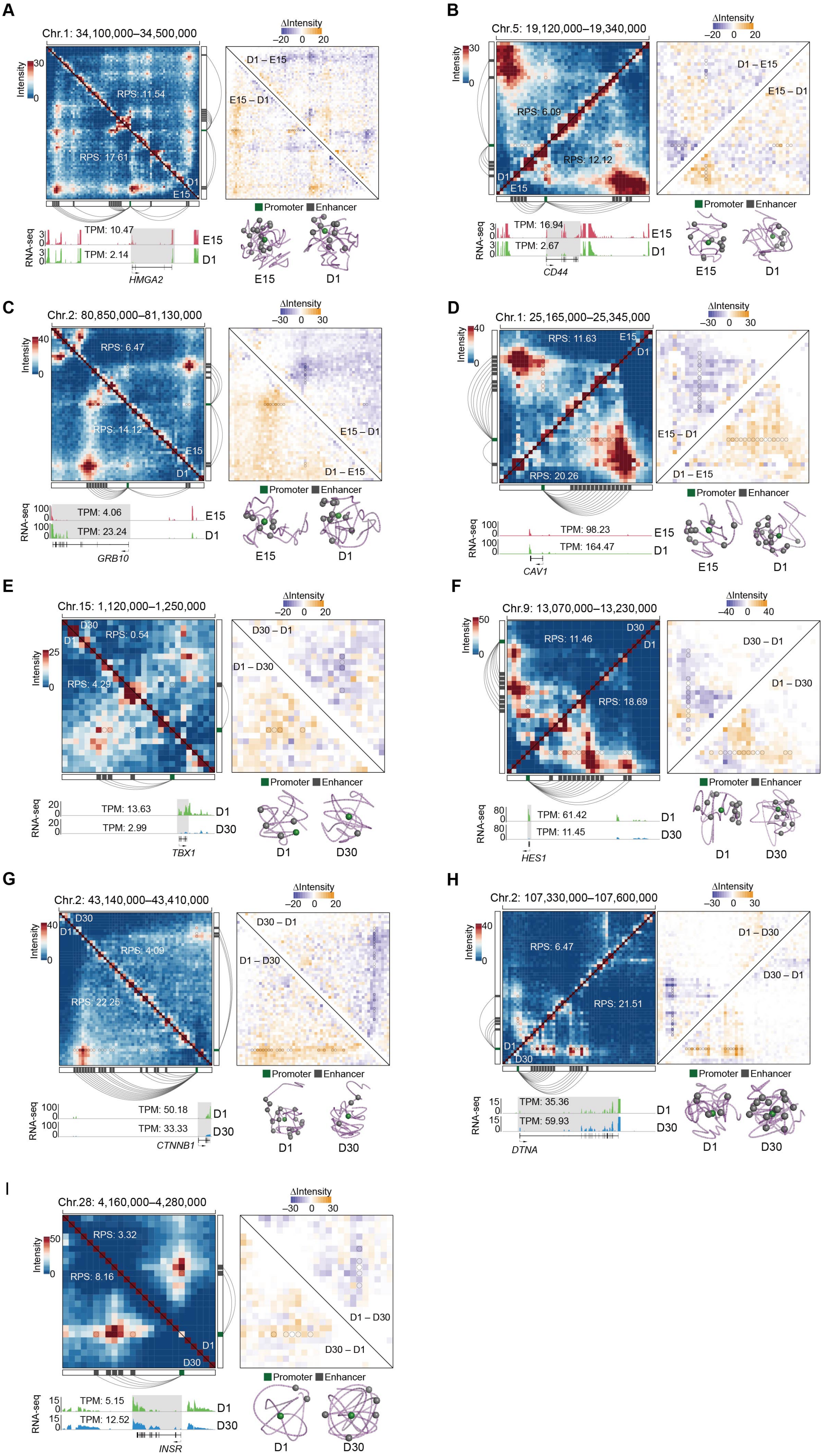
A
Chr.1: 34,100,000–34,500,000
Intensity
30
0
RPS: 11.54
RPS: 17.61
D1
E15
ΔIntensity
–20 0 20
D1 – E15
E15 – D1
Promoter Enhancer
RNA-seq
TPM: 10.47
TPM: 2.14
E15
D1
HMGA2
E15
D1
B
Chr.5: 19,120,000–19,340,000
Intensity
30
0
RPS: 6.09
RPS: 12.12
D1
E15
ΔIntensity
–20 0 20
D1 – E15
E15 – D1
Promoter Enhancer
RNA-seq
TPM: 16.94
TPM: 2.67
E15
D1
CD44
E15
D1
C
Chr.2: 80,850,000–81,130,000
Intensity
40
0
RPS: 6.47
RPS: 14.12
E15
D1
ΔIntensity
–30 0 30
E15 – D1
D1 – E15
Promoter Enhancer
RNA-seq
100
TPM: 4.06
TPM: 23.24
E15
D1
GRB10
E15
D1
D
Chr.1: 25,165,000–25,345,000
Intensity
40
0
RPS: 11.63
E15
D1
RPS: 20.26
ΔIntensity
–30 0 30
E15 – D1
D1 – E15
Promoter Enhancer
RNA-seq
100
TPM: 98.23
TPM: 164.47
E15
D1
CAV1
E15
D1
E
Chr.15: 1,120,000–1,250,000
Intensity
25
0
D30
D1
RPS: 0.54
RPS: 4.29
ΔIntensity
–20 0 20
D30 – D1
D1 – D30
Promoter Enhancer
RNA-seq
20
TPM: 13.63
TPM: 2.99
D1
D30
TBX1
D1
D30
F
Chr.9: 13,070,000–13,230,000
Intensity
50
0
RPS: 11.46
D30
D1
RPS: 18.69
ΔIntensity
–40 0 40
D30 – D1
D1 – D30
Promoter Enhancer
RNA-seq
80
TPM: 61.42
TPM: 11.45
D1
D30
HES1
D1
D30
G
Chr.2: 43,140,000–43,410,000
Intensity
40
0
D30
D1
RPS: 4.09
RPS: 22.25
ΔIntensity
–20 0 20
D30 – D1
D1 – D30
Promoter Enhancer
RNA-seq
100
TPM: 50.18
TPM: 33.33
D1
D30
CTNNB1
D1
D30
H
Chr.2: 107,330,000–107,600,000
Intensity
40
0
RPS: 6.47
RPS: 21.51
D1
D30
ΔIntensity
–30 0 30
D1 – D30
D30 – D1
Promoter Enhancer
RNA-seq
15
TPM: 35.36
TPM: 59.93
D1
D30
DTNA
D1
D30
I
Chr.28: 4,160,000–4,280,000
Intensity
50
0
RPS: 3.32
RPS: 8.16
D1
D30
ΔIntensity
–30 0 30
D1 – D30
D30 – D1
Promoter Enhancer
RNA-seq
15
TPM: 5.15
TPM: 12.52
D1
D30
INSR
D1
D30

Figure S16. PEI rewiring of nine representative genes with differential RPS between neighboring stages. A) *HMGA2* (related to ‘mesoderm formation’). B) *CD44* (related to ‘cellular response to fibroblast growth factor stimulus’). C) *GRB10* (related to 'transmembrane receptor protein tyrosine kinase signalling pathway’). D) *CAV1* (related to ‘regulation of transforming growth factor beta receptor signalling pathway') were identified between E15 and D1. E) *TBX1* (related to ‘TGF-β signaling pathway’). F) *HES1* (related to ‘focal adhesion’). G) *CTNNB1* (related to ‘Hippo signaling pathway’). H) *DTNA* (related to ‘muscle contraction’). I) *INSR* (related to ‘muscle tissue development’) were identified between D1 and D30. From top to bottom: Hi-C maps (upper left), heatmap of interaction intensity differences (upper right), gene expression levels (lower left), and 3D structural models (lower right) of the corresponding genomic regions. Promoters (green squares), enhancers (grey squares), and PEIs (connecting lines) are displayed beside the Hi-C maps.

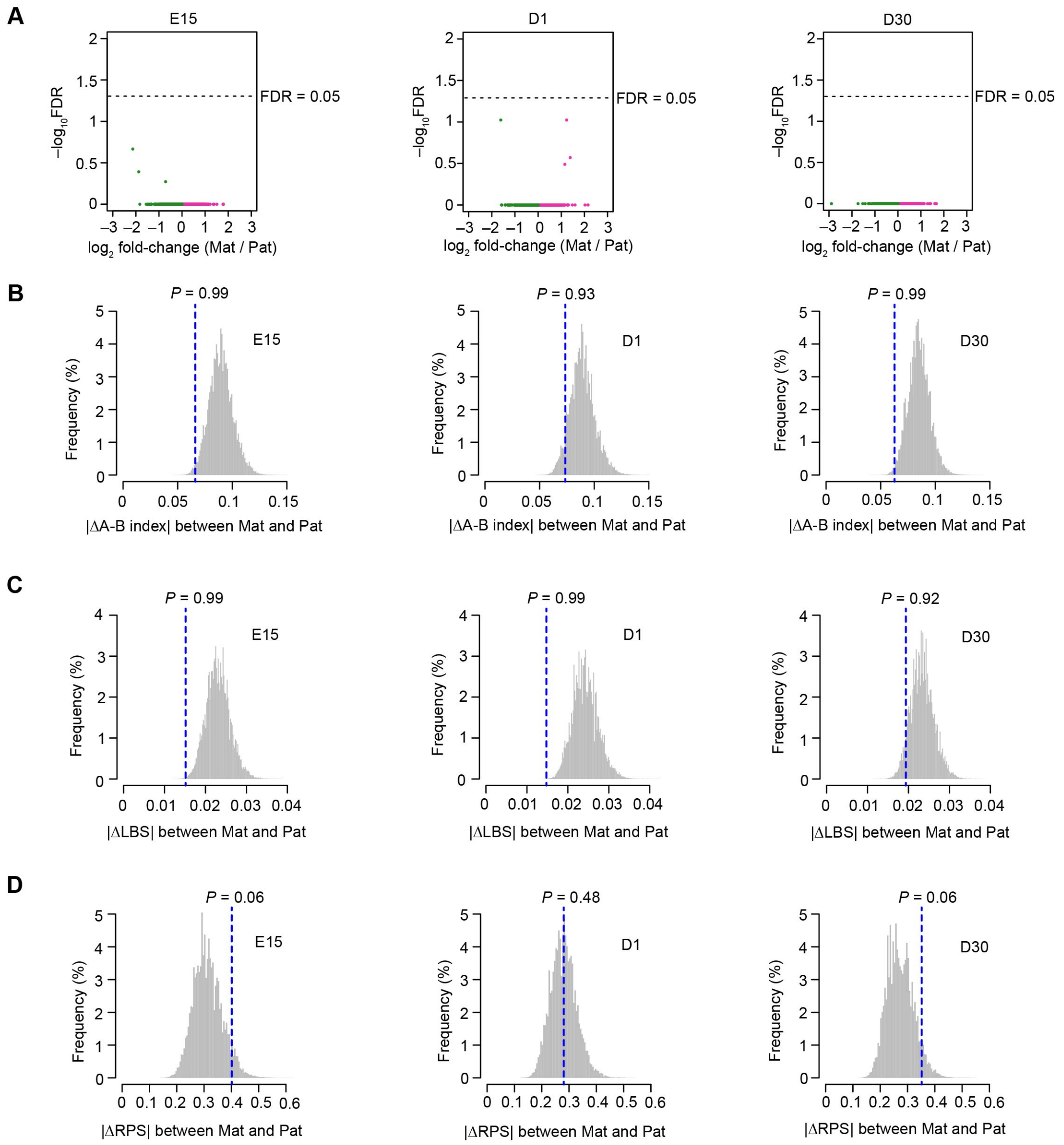

Figure S17. Evaluation of differences in chromatin hierarchical structures between maternal and paternal haplotypes for the 106 chicken orthologs of empirical imprinted genes. A) Volcano plots of allelic gene expression (TPM > 0.5 at either allele) showing no parent-of-origin-specific transcription genome-wide at E15 (left), D1 (middle), and D30 (right), respectively. B–D) Absolute value distribution of A-B index differences (B), LBS differences (C), and RPS differences (D) between maternal and paternal haplotypes in the 100,000 times of randomly sampling of 106 non-imprinted genes at E15 (left), D1 (middle) and D30 (right). The median values of A-B differences for the 106 empirical imprinted genes identified in mammals and with homologs in chicken are indicated by the blue lines. Mat: maternal haplotypes; Pat: paternal haplotypes. *P* values were calculated using ranking probability approach.

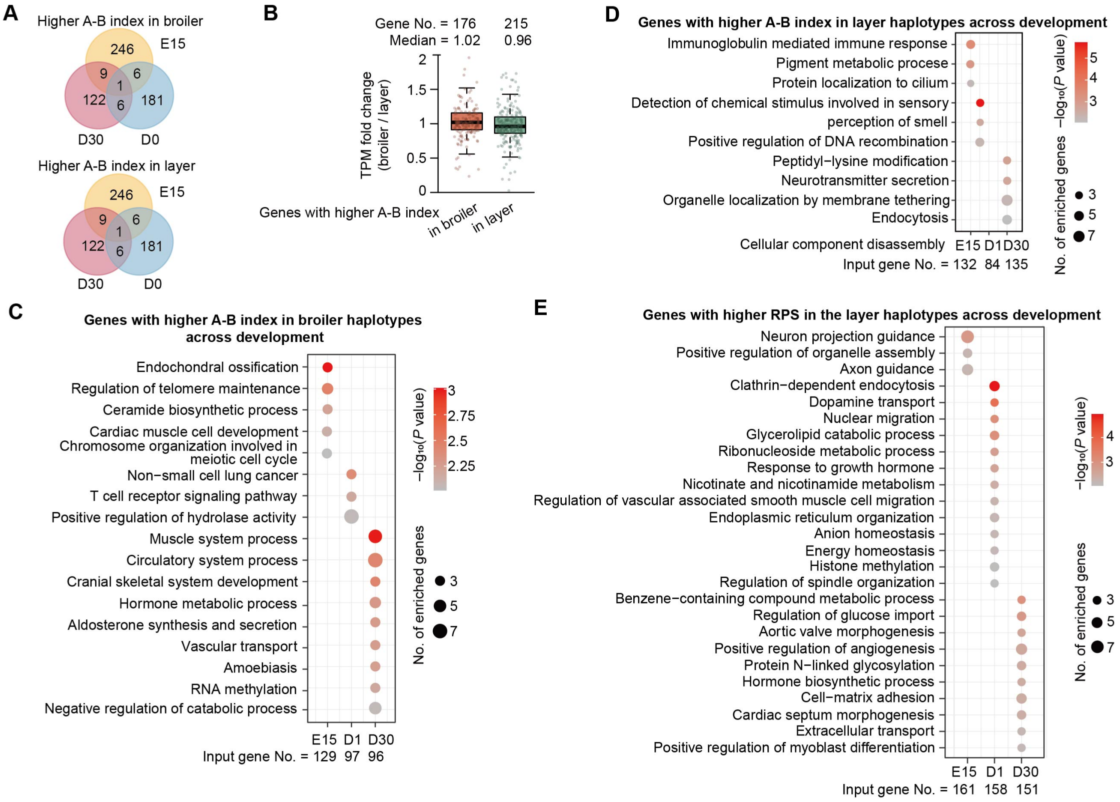

Figure S18. Characteristics of differential compartments between parental breeds in the hybrid chickens. A) Venn plots of 20 kb genomic bins with higher A-B index in either broiler (upper) or layer haplotypes (lower) at the E15, D1, and D30 stage. Number of bins are shown. B) Expression divergence of the genes located in differential compartments between parental breeds. C,D) Outputs of functional enrichment analysis for genes located in more active compartments in either broiler (C) or layer haplotypes (D) across the three developmental stages. The top ten enriched terms are shown. E) Outputs of functional enrichment analysis conducted for the genes with higher RPS in layer haplotypes across the three developmental stages. The top 20 enriched terms are shown.

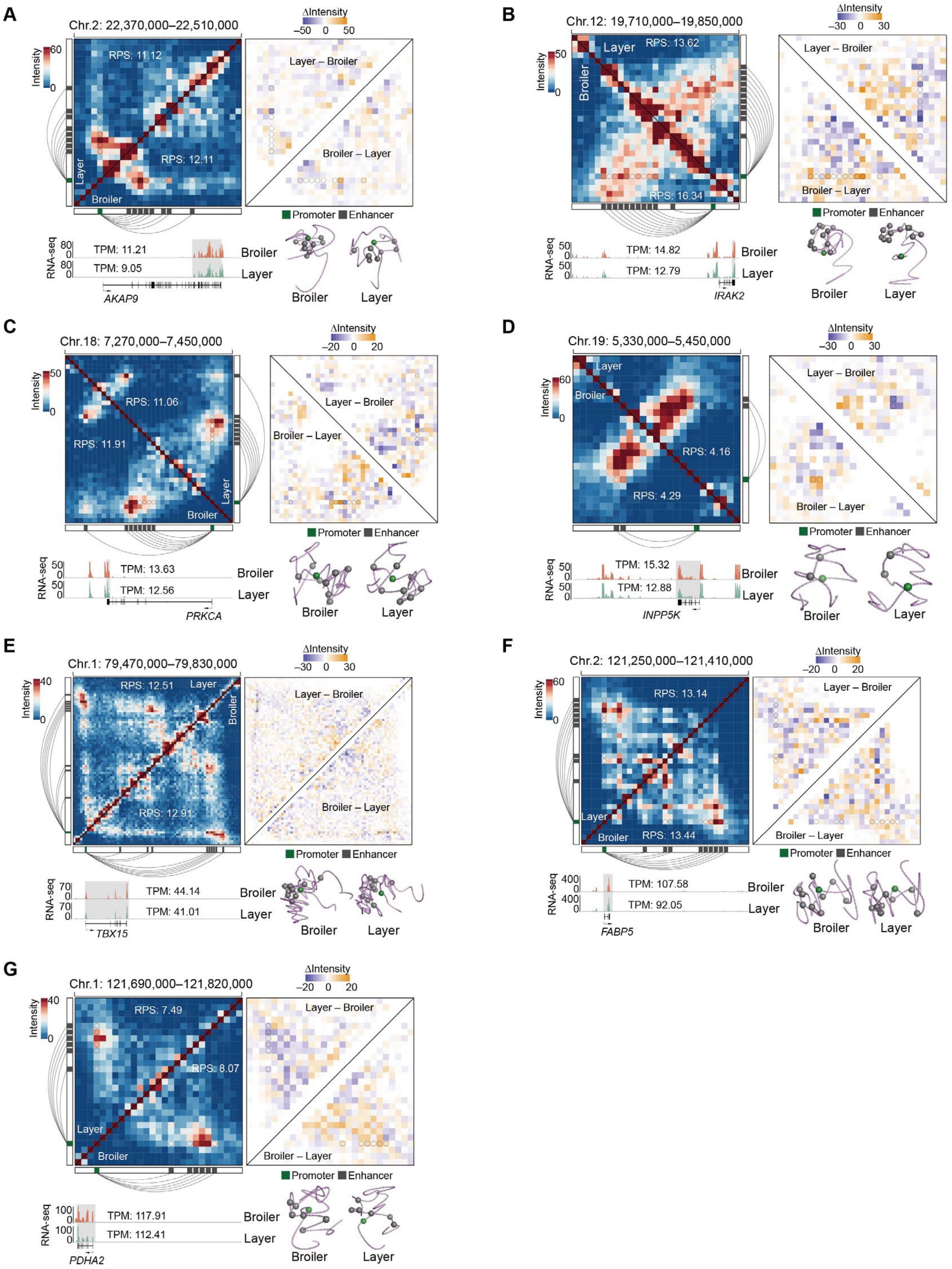

Figure S19. PEI rewiring of representative genes with differential RPS between breeds, including *AKAP9* (the enriched gene in the GO term 'regulation of muscle system process'; A), *IRAK2* (the enriched gene in 'toll-like receptor signalling pathway'; B), and *PRKCA* (the enriched gene in the GO term 'regulation of muscle system process'; C) identified at E15 stage, *INPP5K* (the enriched gene in 'response to growth factor'; D) and *TBX15* (the enriched gene in 'embryo development ending in birth or egg hatching'; E) at D1 stage, *FABP5* and *PDHA2* (the enriched genes in 'acetyl-CoA metabolism'; F,G) at D30 stage. From top to bottom: Hi-C maps (upper left), heatmap of interaction intensity differences (upper right), gene expression levels (lower left) and 3D structural models (lower right) of the corresponding genomic regions. Promoters (green squares), enhancers (grey squares), and PEIs (connecting lines) are displayed beside the Hi-C maps.

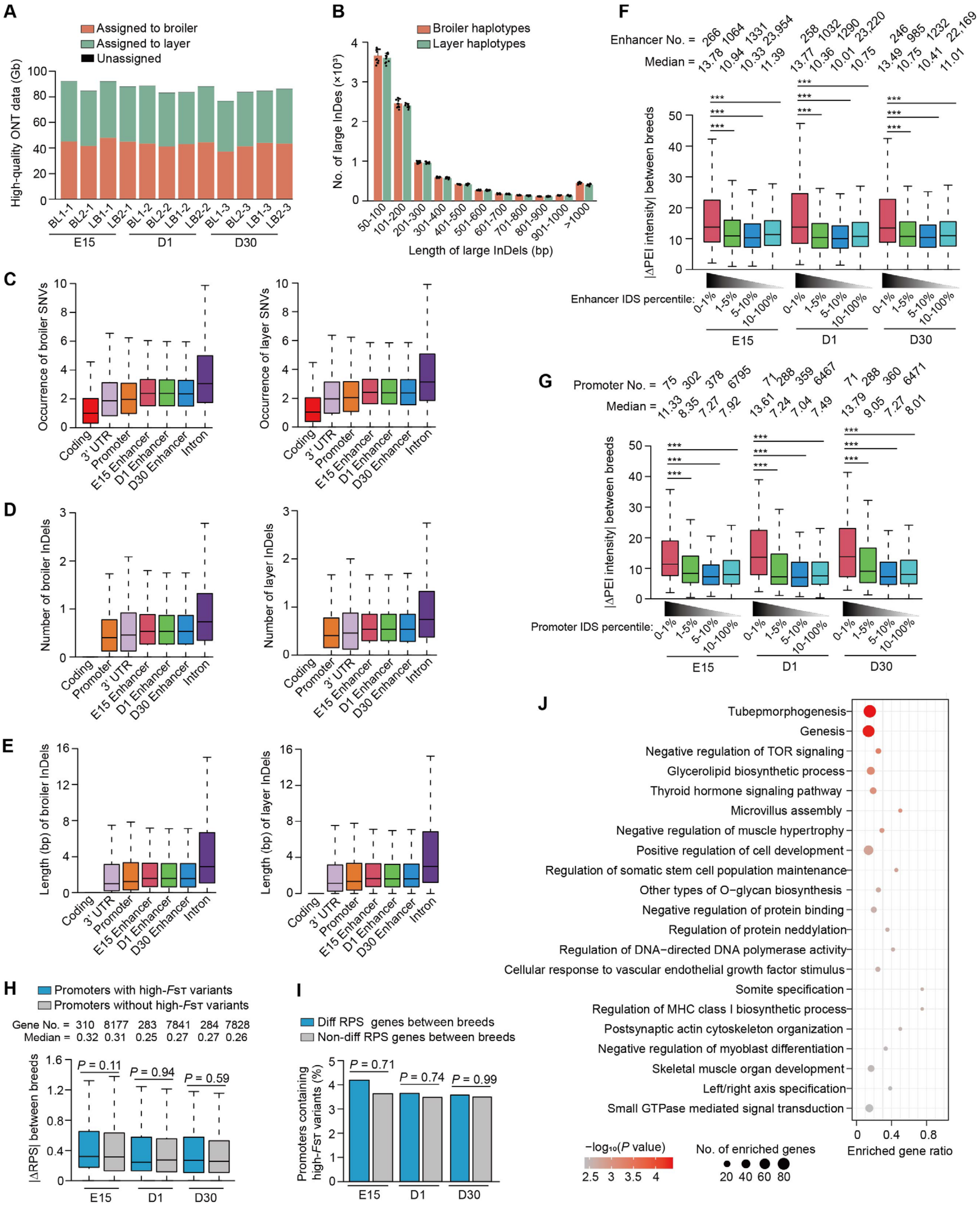

Figure S20. Effects of genomic variants between breeds on PEI wiring in the hybrid chickens. A) Data summary of the high-quality long-read genome sequencing (Oxford Nanopore Technologies) data of F1 hybrid chickens (n = 12). B) Length distribution of the large InDels (≥ 50 bp) identified in the 24 haplotypes of F1 hybrid chickens. Data is shown as means ± SD. The dots represent haplotypes (n = 24). C–E) Distribution frequency of sequence variants in various genomic elements, including the coding (15,636 genes), promoter (15,636 genes), 3'UTR (15,636 genes), enhancer (n = 26,615, 25,800, and 24,632 for E15, D1, and D30, respectively), and intron (14,692 genes) regions. The number of SNVs (C), lengths (D) and numbers of InDels (E), were used to measure the frequencies, respectively. F,G) PEI intensity differences between breeds for the enhancers (F) and the promoters (G) with different levels of sequence divergence (indicated by percentile of identity score (IDS), see Supplementary Methods). It demonstrated that promoters or

enhancers with more sequence variants showed greater differences in PEI intensity between breeds. H) Comparison of RPS difference for genes with promoters carrying versus lacking high-F_{ST} variants. I) Enrichment of promoters with high-F_{ST} variants ($F_{ST} \geq 0.75$) in the differential RPS genes between the parental breeds compared to other genes. J) Outputs of enrichment analysis for the union set of genes (*n* = 1379) having enhancers with high-F_{ST} variants across the three developmental stages. The *P* values were calculated using the Wilcoxon rank-sum test in F–H and Fisher's exact test in I. ** $0.001 < P < 0.01$; *** $P < 0.001$.

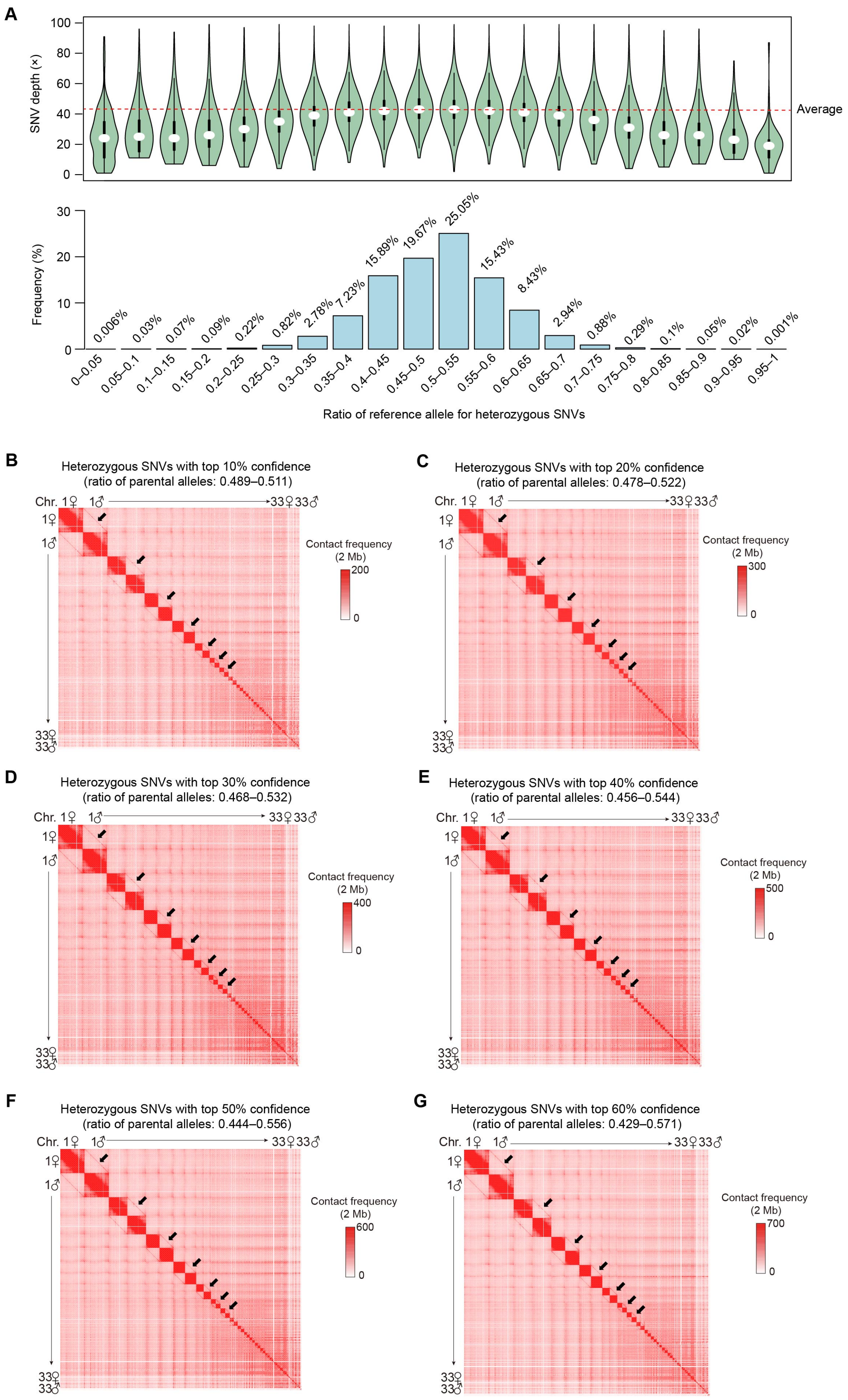
A
SNV depth (×)
Average
Frequency (%)
0.006%
0.03%
0.07%
0.09%
0.22%
0.82%
2.78%
7.23%
15.89%
19.67%
25.05%
15.43%
8.43%
2.94%
0.88%
0.29%
0.1%
0.05%
0.02%
0.001%
0–0.05
0.05–0.1
0.1–0.15
0.15–0.2
0.2–0.25
0.25–0.3
0.3–0.35
0.35–0.4
0.4–0.45
0.45–0.5
0.5–0.55
0.55–0.6
0.6–0.65
0.65–0.7
0.7–0.75
0.75–0.8
0.8–0.85
0.85–0.9
0.9–0.95
0.95–1
Ratio of reference allele for heterozygous SNVs
B
Heterozygous SNVs with top 10% confidence
(ratio of parental alleles: 0.489–0.511)
Contact frequency
(2 Mb)
C
Heterozygous SNVs with top 20% confidence
(ratio of parental alleles: 0.478–0.522)
D
Heterozygous SNVs with top 30% confidence
(ratio of parental alleles: 0.468–0.532)
E
Heterozygous SNVs with top 40% confidence
(ratio of parental alleles: 0.456–0.544)
F
Heterozygous SNVs with top 50% confidence
(ratio of parental alleles: 0.444–0.556)
G
Heterozygous SNVs with top 60% confidence
(ratio of parental alleles: 0.429–0.571)

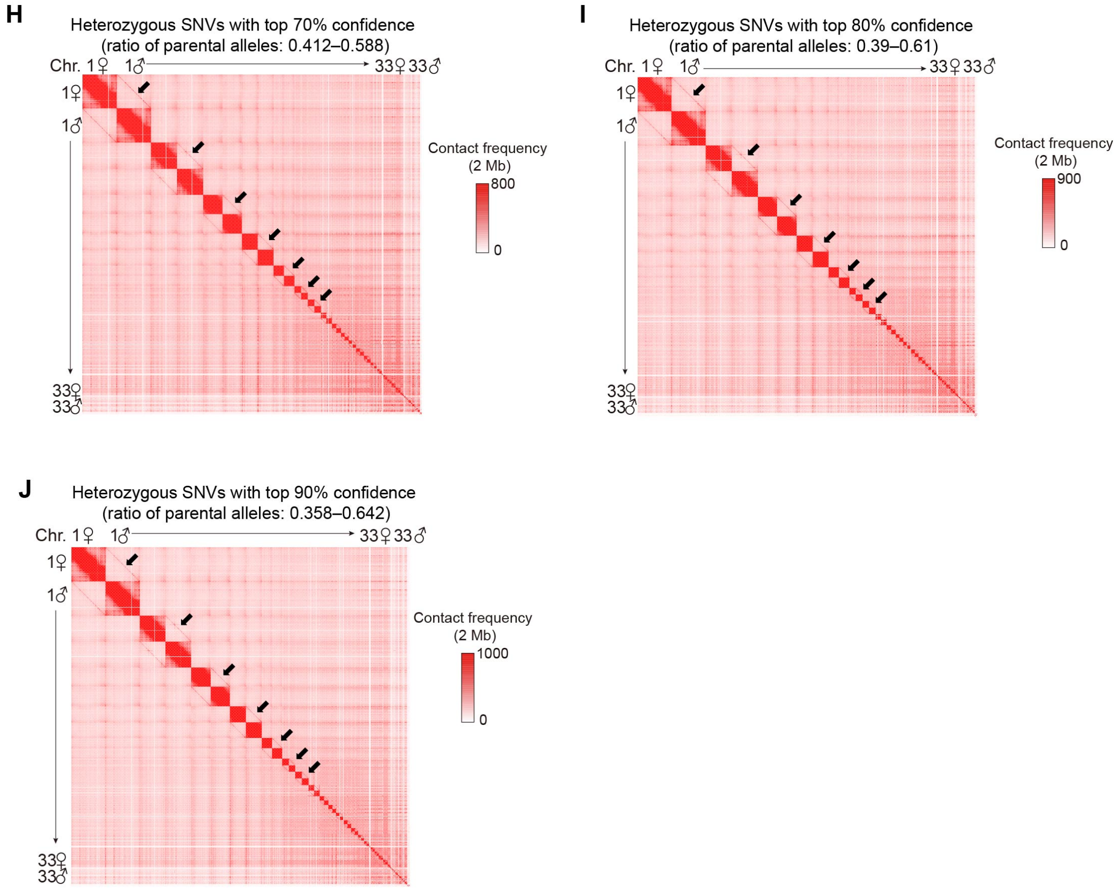

Figure S21. Detection of homolog pairing with heterozygous SNVs at different confidence levels to assign parental origins of chromatin interactions. A) The ratio of parental alleles for each heterozygous SNV. All heterozygous SNVs were divided into 20 intervals. Top: distribution of sequencing depths for SNVs within each interval. Bottom: proportion of SNVs within each interval. B–J) Hi-C maps showing signals of genome-wide homolog pairing (arrows) in a representative hybrid chicken sample based on heterozygous SNVs at different confidence levels.

Supplementary Methods

1. Identification of SNVs and short InDels

We called genome-wide SNVs and short InDels (in genomic variant call format (gVCF)) using the Genome Analysis Toolkit (GATK, v 3.8)[5] HaplotypeCaller. Low-quality variants were filtered out using GATK, with the arguments 'QD < 10.0 || FS > 60.0 || MQ < 40.0 || MQRankSum < –12.5 || ReadPosRankSum < –8.0 || GQ < 30'. Variants located on sex chromosomes, the mitochondrial genome, and unplaced scaffolds were removed from further analyses. We merged the SNVs of all individuals and performed principal component analysis (PCA) using the GCTA software (v 1.93.2)[6] to infer their genetic relationship. To keep as many variants as possible for haplotype phasing, we only combined the variant data of each family (including both parents and three F1 hybrids sampled at three stages) into a multi-sample VCF file using GVCFGenotyper. The variants with the lowest (bottom ~1%) and highest (top ~1%) coverage depth (depth-based Z-score < –2.58 and > 2.58, respectively) were discarded.

2. *In situ* Hi-C library sequencing

Five *in situ* Hi-C libraries were generated as previously described[7] with minor amendments for each of the 12 F1 hybrid chicken skeletal muscle tissue (SMT) samples. In brief, the samples were homogenized with liquid nitrogen and fixed with a 4% formaldehyde solution at room temperature for 30 min. The fixation was quenched using 0.25 $mol \cdot L^{-1}$ glycine for 5 min. The samples were then suspended in a lysis buffer on ice for 15 min. The DNA was digested with 200 U of DpnII (R0543S, NEB, USA) at 37°C for 90 min, 65°C for 20 min and 25°C for 5 min. Next, 0.4 mM Biotin-14-dATP (19524-016, Invitrogen), 10 mM dCTP, 10 mM dGTP, 10 mM dTTP, and 5 $U \cdot \mu L^{-1}$ Klenow Fragment (M0210L, NEB) were used to fill-in the restriction fragment overhangs at 37°C for 45 min. The fragments were ligated by T4 DNA ligase (L6030-HC-L, Enzymatics, USA) at room temperature for 4 hours. The ligated DNA was purified and sheared to a length of 300–500 bp, then washed using M280 beads. The Hi-C libraries were amplified with ten PCR cycles and sequenced with 100 bp paired-end reads on the BGISEQ-500 platform (BGI Inc., Shenzhen, China).

3. Reconstruction of haplotype-resolved Hi-C maps

3.1. Hi-C data mapping

To reduce reference biases and mapping artifacts in the Hi-C data processing, we masked the heterozygous SNVs of F1 hybrids in the chicken reference genome (GRCg6a) with 'N' bases, and high-quality Hi-C reads of F1 hybrid samples were aligned to the variant masked genome. Following the standard pipeline of HiC-Pro (v 2.9.0),[8] experimental Hi-C artefacts and other uninformative di-tags were discarded. In total, 42.51 billion valid contacts were obtained for the Hi-C map construction.

3.2. Building chromosome-level haplotypes

To generate haplotypes as accurate and long as possible, we employed both family genotyping and Hi-C data of F1 hybrids to build haplotype blocks. Briefly, after genotyping of family members using short-read sequencing data, heterozygous SNVs of F1 hybrids can be phased when at least one parent had homozygous genotypes (Figure S3A, Supporting Information). Concurrently, the heterozygous SNVs in Hi-C reads were phased and chromosome-span haplotype blocks were generated using the HapCUT2 (v 1.3.1) (Figure S3B, Supporting Information).[1] By merging the haplotype blocks of both methods which incorporated the vast majority of all heterozygous SNVs (Figure S3C, Supporting Information), we obtained the chromosome-level haplotypes (n = 24) for F1 hybrid chickens.

3.3. Constructing haplotype-resolved Hi-C maps

To incorporate as many Hi-C contacts as possible to draw haplotype-resolved 3D genome maps, we performed haplotype phasing of valid Hi-C contacts mapped on autosomes using SNV phasing, local imputation, and HaploHiC software (v 0.32),[2] based on the number of informative SNVs in each read pair (Figure S2B, Supporting Information). Hi-C reads with informative SNVs on both ends which were recognized by SNPsplit (v 0.3.4),[9] constituting 6.33–7.43% of the valid contacts, can be accurately phased using the well-built haplotypes as mentioned above. Then, the local imputation and HaploHiC program were respectively applied to the Hi-C data with informative SNVs on

one end (partially informative contacts, 37.19–38.83% of the valid contacts) and on neither end (non-informative contacts, 53.86–56.35% of the valid contacts), which was directed by the proportions of intra-homologous (i.e., intra-maternal and intra-paternal), inter-homologous, and inter-heterologous chromosomal contacts within the informative contacts. Of note, when incorporating the large amount of partial and non-informative Hi-C reads, the phasing efficiency was hardly affected by the uneven density of phased SNVs along the genome (Figure S2C, Supporting Information), demonstrating the necessity of using all three classes of contacts in phasing Hi-C reads.

We drew 24 haplotype-resolved intra-chromosomal Hi-C maps and 12 haplotype-resolved inter-chromosomal Hi-C maps using the phased reads. Matrix balancing normalized contact matrices were generated using the Knight-Ruiz (KR) algorithm[10] with the Juicer Tools,[11] which were further normalized across haplotypes using the quantile method[12] and across samples using the counts per million (CPM, normalized to the average abundance across all samples) strategy.

4. Analysis of haplotype-resolved Hi-C maps

4.1. Modelling of diploid 3D genome structures

The 3D genome structures of SMT in hybrid chickens were reconstructed based on the normalized intra- (at 20 kb resolution) and inter-chromosomal (at 500 kb resolution) contact matrices for 32 pairs of homologous autosomes using an approximation of multidimensional scaling (MDS) method implemented with the miniMDS[13] program. The reconstructed models were then visualized using PyMOL (The PyMOL Molecular Graphics System, v 2.5.2 Schrödinger, LLC.).

4.2. Calculation of homolog pairing score (HPS)

To measure the interaction intensity between homologs (i.e., intensity of homolog colocalization), we calculated homolog pairing score (HPS) using the normalized inter-chromosomal Hi-C maps at 20-kb resolution as previously described.[14] The HPS of a 20-kb bin is defined as the $\log_2$-transformed average contact frequency between homologs within the flanking *W* bins up- and downstream of the specific bin:

$$HPS^{w}(i) = log_2(Contact\ Frequency_{m,n})$$

where *m* and *n* correspond to the *i*−*W*-th and *i*+*W*-th bins.

4.3. Identification of A/B compartments

A/B compartments were identified using both PCA and A-B index as previously described[15] with haplotype-resolved intra-chromosomal contact matrices at 20 and 100 kb resolution. Briefly, PCA was performed to generate PC1 vectors at 100-kb resolution. Spearman's *r* between PC1 and genomic features, including GC content and gene density, were then calculated. Compartments A were defined as the bins with positive Spearman's *r*, and compartments B were the remainders. Next, the A-B index was calculated as previously described at 20-kb resolution, assessing the likelihood of a genomic segment interacting with the A or B compartments defined at 100-kb resolution. Then 20 kb bins with positive or negative A-B index were defined as A or B compartments, respectively. The similarity in compartmentalization among haplotypes (n = 24) was assessed using Pearson' *r* of A-B index.

4.4. Detecting variable compartments between haplotypes

We detected compartments with different status (i.e., A or B compartments) or chromatin activities of the same status (i.e., A or more A compartments and B or more B compartments) between haplotypes of different developmental stages, parents of origin, and parental breeds. To detect the compartments with switched status between haplotypes, i.e., A/B switched compartments, we defined the common compartment status of a bin as one that is shared by more than 75% of haplotypes of the same property (e.g., same developmental stage, same parent of origin, and same breed). Compartments of the same status but different chromatin activity, i.e., A/B variable compartments, were identified as the bins with statistically significant differences in A-B index (|ΔA-B index| > 1 for comparison of developmental stages, and |ΔA-B index| > 0.3 for comparison of parental breeds and parents of origin, $P < 0.05$, paired Student's *t*-test).

4.5. Calling of topologically associating domains (TADs)

TADs of 20 kb resolution were identified using the Directionality Index (DI)[15-16] and the Insulation Index (IS)[17] as previously described. Briefly, the DI value

was calculated for each 20 kb bin using the number of Hi-C reads that were mapped from that given bin to the upstream and downstream 2-Mb regions.[16] Domains and anchors were inferred from the DI values using a hidden Markov model (HMM). In addition, the IS value was calculated and normalized for each 20 kb bin. Bins with minimal IS along the normalized IS vector were recognized as the TAD anchors. Finally, large TADs identified by DI were split into small TADs detected based on IS, and then the two sets of haplotype-resolved TADs were merged for further analyses. We employed MoC (assessing the overlap between two TADs by measuring in number of base pairs and considering the overall size of both TADs)[18] and VI (measuring the similarity in all subsets of two TAD structures using a dynamic programming algorithm)[19] to access the similarity in TADs among haplotypes.

4.6. Detection of TAD boundary shifts

TAD boundary was defined as the 20 kb anchor bin along with its 60 kb flanking sequences. Two boundaries were merged as a larger one if they were overlapped. TAD boundary shifts were identified between developmental stages, parents of origin and parental breeds as the changed boundary bins that exhibited significantly differential local boundary scores (LBS), an index reflecting the strength of TAD boundary and calculated as previously described.[20] The detection was performed using a method similar to the identification of differentially methylated regions.[21] Briefly, the bin with statistically differential LBS ($|\Delta LBS| \geq 0.2$, $P < 0.05$, paired Student's *t*-test) between haplotypes was taken as the seed and concatenated to the adjacent bin in the 3' downstream region, then the concatenated bin was detected if a seed bin. The iteration continued until a bin of low variance was joined to smooth the significant difference in LBS. To eliminate 'trailing smear' (i.e., a low-variance bin being incorporated due to very high LBS variance in its preceding bins), the detecting procedure was repeated from 3' to 5' across the genome. The region with different boundary annotations and significantly different LBS between haplotypes and three bins or longer was considered a shifted boundary.

4.7. Identification of promoter-enhancer interactions (PEIs)

High-resolution normalized contact maps allowed for investigation of chromatin conformation at the gene level. Thus, we identified PEIs using the PSYCHIC algorithm[22] with default parameters. The promoter region of a gene was defined as the 5 kb bin covering the transcription start site (TSS). To accelerate the identification, normalized haplotype-resolved Hi-C maps at 5-kb resolution were split into smaller matrices (20 Mb × 20 Mb) with a step size of 10 Mb. High-confidence PEIs were kept according to following parameters: (i) FDR ≤ 10^{-4}; (ii) bridging distance between promoter and enhancer ≥ 25 kb; (iii) more than two occurrences identified in the haplotypes of same parental breed (n = 4) or parent of origin (n = 4) at a stage; and (iv) more than three occurrences identified across all haplotypes of a stage. To explore the regulatory effects of multiple enhancers on a gene, regulatory potential score (RPS) was calculated for each gene as previously reported.[23]

4.8. Detection of differential RPS genes

To detect the genes with evident PEI rewiring, we identified the genes with differential RPS between haplotypes of different developmental stages ($|\Delta RPS| > 3$ and $P < 0.05$), parents of origin ($|\Delta RPS| > 0.3$ and $P < 0.05$), and parental breeds ($|\Delta RPS| > 0.3$ and $P < 0.05$) based on RPS differences and P values calculated using the paired Student's t-test.

5. Quantitation of allelic gene expression

High-quality RNA-seq reads were mapped to the chicken reference genome (GRCg6a) using STAR (v 2.6.0c)[24] with parameters *'--outSAMattributes NH HI NM MD --alignEndsType EndToEnd'*. Total expression level of autosomal protein-coding genes (n = 19,328) was quantified as transcripts per million (TPM) using the Kallisto (v 0.44.0) software.[25] Averagely 88.35% of these autosomal genes had phased exonic SNVs and can be parsed allelic expression using Allelome.PRO.[26] For genes with evident transcription above the threshold of ≥ 0.5 TPM, the allelic expression was determined as the product of the maternal against paternal allelic read ratios and the total TPM for both alleles when there were 10 or more mapped reads with assigned parents

of origin. For genes without evident transcription (TPM < 0.5), their allelic expressions were set as equal.

6. Long-read genome sequencing

Long-read sequencing (Oxford Nanopore Technologies, ONT) was performed on the F1 hybrid chickens (*n* = 12). The genomic DNA was extracted from the fetal (E15) or liver (D1 and D30) tissue using a proteinase K/phenol:chloroform extraction method.[27] DNA quality and concentration were verified by 1.0% agarose gel electrophoresis and Qubit 4 (Thermo Fisher Scientific, Waltham, MA, USA), respectively. Libraries for ONT PromethION sequencing were prepared using the ligation sequencing kit (Oxford Nanopore Technologies, New York, NY, USA: SQK-LSK109) following the manufacturer's instructions. The products were quantified using the Bioanalyzer 2100 (Agilent, Santa Clara, CA, USA), and raw signals were generated by the PromethION R9.4 platform. Base-calling from the raw signal data was carried out using Guppy v4.0.11/MinKNOW v19.05.1 with the High-accuracy model (Oxford Nanopore Technologies, UK).

7. Identification of large insertions and deletions (InDels)

Clean long-read sequencing (ONT) data was filtered by discording reads of low-quality or shorter than 1 kb. We used short reads from two parental haplotypes to first partition long reads from an offspring into haplotype-specific sets based on the presence of the haplotype-specific *k*-mers using a trio binning method[28] implemented with the TrioCanu module of the Canu assembler. Then the haplotype-resolved long reads were then error-corrected using the NECAT software (v 0.0.1)[29] and mapped to the chicken reference genome using the software NGMLR (v 0.2.7)[30] with default parameters. The uniquely aligned reads were used for identification of large InDels (≥ 50 bp in length) with the software Sniffles (v 1.0.11).[30] High-confidence large InDels in all haplotypes that were covered by at least ten reads were merged using the software SURVIVOR.[31]

8. Effects of genetic variants on PEIs

8.1. Calculation of the identity score (IDS)

To measure the degree of sequence divergence among haplotypes, we calculated the identity score (IDS) for the given regions as followings:

$$IDS = 1 - (Diff_{nuc}/Total_{nuc})$$

where the $Diff_{nuc}$ means the number of different nucleotides (including SNVs, short and large InDels) between the two haplotypes. $Total_{nuc}$ was defined as the total nucleotide number for a given region. Notably, insertions occurring in either haplotype increased the value of $Total_{nuc}$, whereas deletions occurring in both haplotypes had an opposite effect.

8.2. Calculation of *F*-statistics (F_{ST})

To measure the sequence divergence at the population level between the broiler and layer breeds, we sequenced ~41.94× (44.68 Gb) whole-genome sequencing data for additional purebred broilers (n = 8) and layers (n = 8). Combined with the genome sequencing data of eight parental individuals (four broilers and four layers) used in the reciprocal crosses, we called SNVs and short InDels at the population level and inferred the genetic differentiation (F_{ST}) between the two breeds using vcftools (v 0.1.15)[32] for each phased variant identified in the F1 hybrids. For large InDels called by long-read DNA sequencing, we first inferred the genotype in each purebred individual using the paragraph software (v 2.4a)[33] and then calculated F_{st} using vcftools.

References

[1] P. Edge, V. Bafna, V. Bansal, *Genome Res.* **2017**, *27*, 801.

[2] S. Lindsly, W. Jia, H. Chen, S. Liu, S. Ronquist, C. Chen, X. Wen, C. Stansbury, G. A. Dotson, C. Ryan, *iScience* **2021**, *24*, 103452.

[3] a) F. Berti, J. M. Nogueira, S. Wöhrle, D. R. Sobreira, K. Hawrot, S. Dietrich, *J. Anat.* **2015**, *227*, 361; b) J. Chal, O. Pourquié, *Development* **2017**, *144*, 2104.

[4] M. Mohammadabadi, F. Bordbar, J. Jensen, M. Du, W. Guo, *Animals* **2021**, *11*, 835.

[5] A. McKenna, M. Hanna, E. Banks, A. Sivachenko, K. Cibulskis, A. Kernytsky, K. Garimella, D. Altshuler, S. Gabriel, M. Daly, *Genome Res.* **2010**, *20*, 1297.

[6] J. Yang, S. H. Lee, M. E. Goddard, P. M. Visscher, *Am. J. Hum. Genet.* **2011**, *88*, 76.

[7] S. S. Rao, M. H. Huntley, N. C. Durand, E. K. Stamenova, I. D. Bochkov, J. T. Robinson, A. L. Sanborn, I. Machol, A. D. Omer, E. S. Lander, *Cell* **2014**, *159*, 1665.

[8] N. Servant, N. Varoquaux, B. R. Lajoie, E. Viara, C.-J. Chen, J.-P. Vert, E. Heard, J. Dekker, E. Barillot, *Genome Bio.* **2015**, *16*, 1.

[9] F. Krueger, S. R. Andrews, *F1000Research* **2016**, *5*.

[10] P. A. Knight, D. Ruiz, *IMA J. Numer. Anal.* **2013**, *33*, 1029.

[11] N. C. Durand, M. S. Shamim, I. Machol, S. S. Rao, M. H. Huntley, E. S. Lander, E. L. Aiden, *Cell Syst.* **2016**, *3*, 95.

[12] K. Fletez-Brant, Y. Qiu, D. U. Gorkin, M. Hu, K. D. Hansen, *BioRxiv* **2017**, 214361.

[13] L. Rieber, S. Mahony, *Bioinformatics* **2017**, *33*, i261.

[14] J. AlHaj Abed, J. Erceg, A. Goloborodko, S. C. Nguyen, R. B. McCole, W. Saylor, G. Fudenberg, B. R. Lajoie, J. Dekker, L. A. Mirny, *Nat. Commun.* **2019**, *10*, 4485.

[15] M. J. Rowley, M. H. Nichols, X. Lyu, M. Ando-Kuri, I. S. M. Rivera, K. Hermetz, P. Wang, Y. Ruan, V. G. Corces, *Mol. Cell.* **2017**, *67*, 837.

[16] J. R. Dixon, S. Selvaraj, F. Yue, A. Kim, Y. Li, Y. Shen, M. Hu, J. S. Liu, B. Ren, *Nature* **2012**, *485*, 376.

[17] E. Crane, Q. Bian, R. P. McCord, B. R. Lajoie, B. S. Wheeler, E. J. Ralston, S. Uzawa, J. Dekker, B. J. Meyer, *Nature* **2015**, *523*, 240.

[18] M. Zufferey, D. Tavernari, E. Oricchio, G. Ciriello, *Genome Bio.* **2018**, *19*, 1.

[19] N. Sauerwald, C. Kingsford, *Bioinformatics* **2018**, *34*, i475.

[20] Z. Han, K. Cui, K. Placek, N. Hong, C. Lin, W. Chen, K. Zhao, W. Jin, *Genome Res.* **2020**, *30*, 1097.

[21] M. Li, H. Wu, Z. Luo, Y. Xia, J. Guan, T. Wang, Y. Gu, L. Chen, K. Zhang, J. Ma, *Nat. Commun.* **2012**, *3*, 850.

[22] G. Ron, Y. Globerson, D. Moran, T. Kaplan, *Nat. Commun.* **2017**, *8*, 2237.

[23] M. Zhi, J. Zhang, Q. Tang, D. Yu, S. Gao, D. Gao, P. Liu, J. Guo, T. Hai, J. Gao, *Cell Res.* **2022**, *32*, 383.

[24] A. Dobin, C. A. Davis, F. Schlesinger, J. Drenkow, C. Zaleski, S. Jha, P. Batut, M. Chaisson, T. R. Gingeras, *Bioinformatics* **2013**, *29*, 15.

[25] N. L. Bray, H. Pimentel, P. Melsted, L. Pachter, *Nat. Biotechnol.* **2016**, *34*, 525.

[26] D. Andergassen, C. P. Dotter, T. M. Kulinski, P. M. Guenzl, P. C. Bammer, D. P. Barlow, F. M. Pauler, Q. J. Hudson, *Nucleic Acids Res.* **2015**, *43*, e146.

[27] S. J. a. R. D. W., *Molecular cloning: a laboratory manual, 3rd edition*, *Cold Spring Harbor Laboratory Press*. **2001**.

[28] S. Koren, A. Rhie, B. P. Walenz, A. T. Dilthey, D. M. Bickhart, S. B. Kingan, S. Hiendleder, J. L. Williams, T. P. Smith, A. M. Phillippy, *Nat. Biotechnol.* **2018**, *36*, 1174.

[29] Y. Chen, F. Nie, S.-Q. Xie, Y.-F. Zheng, Q. Dai, T. Bray, Y.-X. Wang, J.-F. Xing, Z.-J. Huang, D.-P. Wang, *Nat. Commun.* **2021**, *12*, 60.

[30] F. J. Sedlazeck, P. Rescheneder, M. Smolka, H. Fang, M. Nattestad, A. Von Haeseler, M. C. Schatz, *Nat. Methods.* **2018**, *15*, 461.

[31] D. C. Jeffares, C. Jolly, M. Hoti, D. Speed, L. Shaw, C. Rallis, F. Balloux, C. Dessimoz, J. Bähler, F. J. Sedlazeck, *Nat. Commun.* **2017**, *8*, 14061.

[32] P. Danecek, A. Auton, G. Abecasis, C. A. Albers, E. Banks, M. A. DePristo, R. E. Handsaker, G. Lunter, G. T. Marth, S. T. Sherry, *Bioinformatics* **2011**, *27*, 2156.

[33] S. Chen, P. Krusche, E. Dolzhenko, R. M. Sherman, R. Petrovski, F. Schlesinger, M. Kirsche, D. R. Bentley, M. C. Schatz, F. J. Sedlazeck, *Genome Bio.* **2019**, *20*, 1.
